# A scoping review of artificial intelligence-based methods for diabetes risk prediction

**DOI:** 10.1038/s41746-023-00933-5

**Published:** 2023-10-25

**Authors:** Farida Mohsen, Hamada R. H. Al-Absi, Noha A. Yousri, Nady El Hajj, Zubair Shah

**Affiliations:** 1 College of Science and Engineering, Hamad Bin Khalifa University, Qatar Foundation, 34110 Doha, Qatar; 2 Genetic Medicine, Weill Cornell Medicine-Qatar, Qatar Foundation, Doha, Qatar; 3 College of Health and Life Sciences, Hamad Bin Khalifa University, Qatar Foundation, 34110 Doha, Qatar; 4 Computer and Systems Engineering, Alexandria University, Alexandria, Egypt

**Keywords:** Type 2 diabetes, Preventive medicine

## Abstract

The increasing prevalence of type 2 diabetes mellitus (T2DM) and its associated health complications highlight the need to develop predictive models for early diagnosis and intervention. While many artificial intelligence (AI) models for T2DM risk prediction have emerged, a comprehensive review of their advancements and challenges is currently lacking. This scoping review maps out the existing literature on AI-based models for T2DM prediction, adhering to the PRISMA extension for Scoping Reviews guidelines. A systematic search of longitudinal studies was conducted across four databases, including PubMed, Scopus, IEEE-Xplore, and Google Scholar. Forty studies that met our inclusion criteria were reviewed. Classical machine learning (ML) models dominated these studies, with electronic health records (EHR) being the predominant data modality, followed by multi-omics, while medical imaging was the least utilized. Most studies employed unimodal AI models, with only ten adopting multimodal approaches. Both unimodal and multimodal models showed promising results, with the latter being superior. Almost all studies performed internal validation, but only five conducted external validation. Most studies utilized the area under the curve (AUC) for discrimination measures. Notably, only five studies provided insights into the calibration of their models. Half of the studies used interpretability methods to identify key risk predictors revealed by their models. Although a minority highlighted novel risk predictors, the majority reported commonly known ones. Our review provides valuable insights into the current state and limitations of AI-based models for T2DM prediction and highlights the challenges associated with their development and clinical integration.

## Introduction

Precision diabetes medicine represents a cutting-edge approach to diagnosing, predicting, and treating diabetes. This approach accounts for individual variations and integrates diverse data sources to comprehensively understand an individual’s health status, predisposition, and treatment response^1,2^. Type 2 diabetes mellitus (T2DM) is the most prevalent form of diabetes, and its global incidence and prevalence are growing, putting a significant burden on healthcare systems. Given the economic and personal impact of T2DM, including decreased productivity, higher healthcare costs, severe complications, and shortened lifespan, there is a pressing need for preventive efforts.

Precision prognostics, a critical aspect of precision diabetes medicine, aims to develop predictive models to estimate an individual’s risk of developing T2DM and its complications based on their risk profiles^1^. This enables the identification of high-risk individuals, allowing for personalized prevention strategies and targeted treatments to delay or prevent the onset of the disease and its complications^1,3^. The American Diabetes Association (ADA) and the European Association for the Study of Diabetes (EASD) Consensus Report support this approach and recommend targeting high-risk individuals with lifestyle interventions and glucose-lowering medications to prevent or delay the onset of T2DM^1^.

For prognostic models to be implemented into routine care, they must go through different stages, including model development, evaluation, and translation to clinical decision support^4–6^. The development of these models entails utilizing longitudinal data that reflect individuals’ biological characteristics, lifestyle, and environmental interactions^7^. The next crucial phase involves evaluating the model’s performance. An effective predictive model is characterized by its ability to accurately estimate an individual’s risk—where predictions align closely with observed outcomes (calibration), its ability to reliably distinguish between individuals at high and low risk of developing the condition (discrimination), and its effectiveness across diverse populations (generalizability)^8^. Both calibration and discrimination can be assessed either through internal validation (using the same dataset on which the model was developed) or external validation (employing a different dataset), with external validation often preferred as it provides a more comprehensive assessment of the model’s generalizability^7,9^.

Many researchers have proposed T2DM risk prediction models, often in the form of risk scores^8,10^. These models, however, have limitations. They often use a limited number of risk factors as input features, which does not consider the complex interplay among different biological systems involved in the development of T2DM^10,11^. Additionally, such models often rely heavily on previous literature for predictor selection. This reliance may limit the model’s scope, potentially overlooking novel or less-explored predictors and thus not fully capturing the complexity of T2DM pathogenesis^12^.

Recently, artificial intelligence (AI), particularly machine learning (ML), and deep learning (DL), has attracted increasing attention in medical research due to its capability to analyze large biomedical datasets, including electronic health records (EHRs), medical imaging, multi-omics data, behavioral/wellness, and environmental data^13,14^. AI-driven models have emerged as a promising tool for developing predictive models for T2DM by analyzing complex and multidimensional datasets to identify high-risk individuals, uncover risk factors and biomarkers associated with T2DM development, and guide personalized interventions for disease prevention. While most existing AI-based T2DM predictive models focus on a single modality of data (i.e., EHR), there has been a recent shift toward multimodal models integrating different data modalities.

As the landscape of AI-based T2DM predictive models is rapidly evolving, there is a clear need for a comprehensive overview of the relevant literature. To our knowledge, a limited number of reviews have explored the application of AI techniques in diabetes. Notably, these existing reviews differ from our current study in terms of their scope. For instance, one study conducted a meta-analysis of the predictive ability of ML models for T2DM risk prediction but was restricted by a limited sample size of 12 studies^10^. Other reviews focused on evaluating AI models for diabetes detection^15,16^ or predicting diabetes-related complications^17^. Our scoping review, however, focuses on studies that utilize AI-based methods for T2DM risk prediction, particularly those harnessing longitudinal data to construct their predictive models. Our review spans a wide range of AI models, from unimodal to multimodal approaches, and includes 40 studies. These studies encompass various data modalities, including EHRs, multi-omics, and imaging data. Table 1 highlights the main differences between our scoping review and previous reviews in the field, emphasizing the need for a new and updated review.

**Table 1:** Literature review comparison.

Previous reviews	Year	Scope	Comparative contribution of our review
Predictive ability of current machine learning algorithms for type 2 diabetes mellitus: a meta-analysis^10^	2022	They conducted a review with meta-analysis to evaluate the current ability of ML algorithms for T2DM prognosis retrieving 12 studies till 2020. All the retrieved studies used EHR data.	Our review focuses on the use of AI models for T2DM risk prediction, with a particular emphasis on studies that used longitudinal data. Moreover, our review covered more studies (*n* = 40) with a wider range of data modalities, including EHR, multi-omics, and imaging.
Machine learning and artificial intelligence-based Diabetes Mellitus detection and self-management: a systematic review^15^	2022	Their review focused on the use of AI models for diabetes detection.	Our review focuses on the use of AI models for T2DM risk prediction, with a particular emphasis on studies that used longitudinal data.
Use and performance of machine learning models for type 2 diabetes prediction in community settings: a systematic review and meta-analysis^16^	2020	They assessed ML’s discrimination ability to predict and diagnose T2DM, covering only eight studies on T2DM risk prediction.	Our review focuses on the use of AI models for T2DM risk prediction, with a particular emphasis on studies that used longitudinal data. Moreover, our review covered more studies with a wider range of AI models for T2DM prognosis, including unimodal and multimodal models.
Microvascular complications in type-2 diabetes: a review of statistical techniques and machine learning models^17^	2020	They conducted a review on microvascular complications in diabetes (retinopathy, neuropathy, nephropathy).	Our review focuses on AI models for T2DM risk prediction rather than its complications.

The primary aim of our review is to explore and provide a comprehensive analysis of the use of AI-based models for T2DM risk prediction. This involves examining the various AI models utilized, the types of data and predictors employed, the datasets and evaluation metrics used, as well as the risk predictors reported that can guide preventive and early intervention strategies. Additionally, we critically evaluate the limitations of AI models in this context and highlight the challenges associated with their clinical implementation. Our review also aims to identify knowledge gaps in the field, highlighting areas where further research is needed to advance the application of AI in T2DM risk prediction.

## Results

This section provides a comprehensive overview of the findings from our scoping review, organized around several key themes (subsections) that emerged during our analysis. We begin with the study selection process and a detailed discussion of the characteristics of the included studies. Next, we describe the data used in these studies, including data modalities, resources, study populations, sample size, participants’ demographics, data imbalance, and missing data handling. In the subsequent subsection, we discuss the AI-based models and methodologies applied, differentiating between unimodal and multimodal models. This is followed by a section detailing validation procedures, performance metrics, and performance comparison between unimodal and multimodal models. The penultimate subsection is dedicated to the studies’ interpretation and reported risk predictors. Finally, we examine the reporting standards and reproducibility of the included studies.

### Study selection and characteristics

**Fig. 1:**
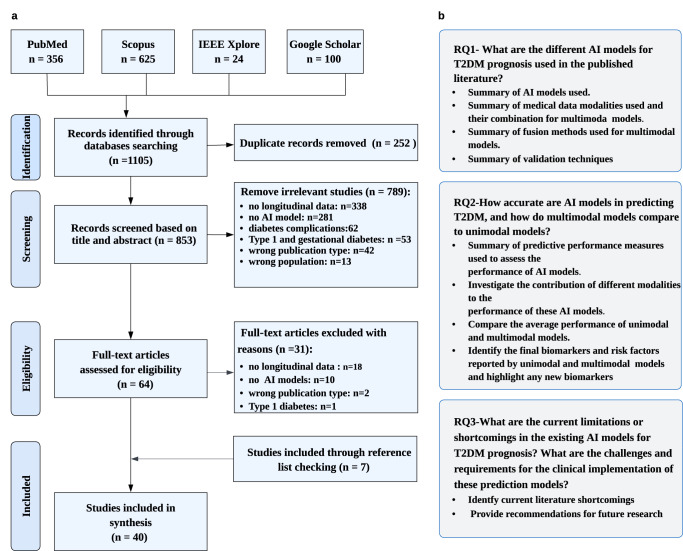
Overview of the study selection process and research questions. **a** Flow diagram illustrating the PRISMA approach for the identification, screening, and selection of studies. **b** Research questions posed.

After an initial screening based on titles and abstracts, 64 studies were considered eligible for full-text screening. For various reasons, 31 studies were excluded after a thorough full-text review against established inclusion criteria. Additionally, seven studies were identified through forward and backward reference screening, resulting in a total of 40 studies that underwent data extraction and synthesis. The overall study screening and selection process is depicted in Fig. 1a. In addition, the characteristics of the included studies are presented in Supplementary Tables 4 and 5.

**Fig. 2:**
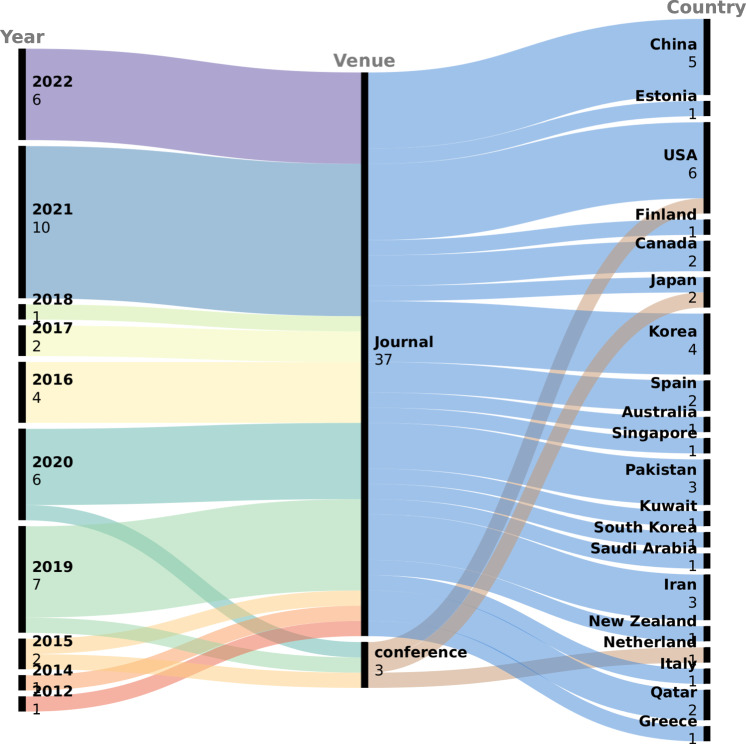
Publication trends in AI-based T2DM prediction. This figure illustrates the distribution of studies based on the publication type, year, and country of publication.

Among the included studies, 37 were peer-reviewed journal articles, and 3 were conference publications. The studies were published between 2012 and 2022, with the majority being published in the past 4 years (*n* = 29). This suggests a growing interest in using AI models for T2DM prognosis, which can be attributed to advancements in AI models and the increasing focus on precision medicine. The studies were conducted in a diverse range of countries, with the majority being from the United States (*n* = 6), followed by China (*n* = 5) and Korea (*n* = 4). The diversity in the country of publication highlights the global interest in using AI models for T2DM prognosis. The distribution of studies by publication type, year, and country of publication is presented in Fig. 2.

All of the AI prediction models included in this scoping review were developed using data from longitudinal cohort studies in accordance with the inclusion criteria outlined in this study. The study designs varied among the included studies, with the majority being retrospective cohort studies (*n* = 18)^11,18–34^ that utilized data from large clinical databases and registries. Other study designs included prospective cohort studies (*n* = 10)^35–44^, case–control studies (*n* = 11)^45–55^, and one case–cohort study^56^. In terms of prediction horizon, the studies reported a wide range of horizons, with most of the studies focusing on medium-term predictions (*n* = 18)^18,18,20–22,24,29,31,37–39,42–45,49,50,56^, such as predicting diabetes onset within 5–10 years. Additionally, 13 studies^22,26–28,32,34–36,48,52–55^ focused on short-term predictions (<5 years), and eight studies predicted T2DM onset within 5 years^20,22,23,25,30,33,36,45^. A smaller number of studies (*n* = 5)^19,40,41,46,47^ reported long-term predictions 10 years prior to T2DM onset. It is worth noting that some studies reported multiple prediction horizons; therefore, the sum of the studies in all categories may not equal the total number of studies included in this review.

Regarding AI applications for T2DM risk prediction, the studies in this review mostly focused on four key areas. The first and most prevalent area of focus was the development of custom predictive models (*n* = 25). These studies primarily addressed the need for accurate, timely, and potentially personalized risk predictions for T2DM^11,20,22,23,25,26,28,30–35,37–40,43–45,47,51,52,54,55^. The target populations, prediction horizons, and data types used varied among these studies. The second key area focused on risk stratification, aiming to identify individuals at high risk of T2DM, an essential component for effective public health intervention^27,29,41,42,49,50,53^. Thirdly, several studies focused on advancing the understanding of disease pathophysiology by identifying new risk predictors or evaluating the prognostic value of certain markers, which could reveal new pathways for prevention and treatment^18,19,24,36,46,48,56^. Finally, two studies focused on the evaluation and comparison of different predictive models^21,37^. Such studies aimed to validate existing models or compare traditional statistical methods with ML approaches. See Supplementary Table 4 for a detailed breakdown of the studies’ aims.

The included studies in our review followed diverse procedures for labeling outcome variables and ascertaining T2DM diagnosis, as shown in Supplementary Table 4. Fasting plasma glucose (FPG) levels of ≥7.0 mmol/L or ≥126 mg/dL and Hemoglobin A1c (HbA1c) of ≥6.5% were the most commonly used criteria. These were used, either independently or in combination, in several studies^11,18,19,23,26,32,33,37,39–42,44,46,49,52,54–56^. The use of medication history or current medication usage for T2DM was also common across numerous studies^11,18,20,23,27,35,37,39–41,44,45,56^. Three studies considered a 2-h blood glucose (2h-BG) level after a 75g Oral Glucose Tolerance Test (OGTT) of ≥200 mg/dL (11.1 mmol) as part of their diagnostic approach^18,24,42^. Self-reported T2DM was another diagnostic criterion used in eight studies^11,20,26,32,35,43,44,47^. International Classification of Diseases codes, specifically ICD-9 and ICD-10, were also utilized in some studies^27,34,45,52^. One study utilized clinical procedures and validated them using recorded blood glucose levels^22^. However, diagnostic methods were not available for some studies^21,25,28–30,36,38,50,51,53^.

### Data

In the development of AI models for predicting T2DM risk, data modalities, and resources are critical aspects to consider. This review revealed a broad range of data modalities in the included studies, highlighting the diversity of information predicting T2DM. EHRs were used by all 40 studies, including sociodemographic characteristics, family history of diabetes (FHD) and other diseases, lifestyle risk factors, anthropometric measures, glycemic traits, blood lipids, blood pressure factors, etc. Other sources incorporated multi-omics data, such as single nucleotide polymorphisms (SNPs), metabolomic data in the form of metabolite levels in the blood, and microbiome data. Additionally, medical imaging data from modalities such as computed tomography (CT) and fundus images were utilized. Notably, several studies fused different modalities to create multimodal predictive models, predominantly combining EHR with multi-omics or medical imaging data. Table 2 summarizes the data modalities and their combinations used in these studies.

**Table 2:** Data types used by the included studies.

Data type	Number of studies	Study reference
EHR	*n* = 40	^11,18–55,84^
EHR+ genetics	*n* = 2	^35,47^
EHR+ metabolomics	*n* = 2	^24,48^
EHR+ metabolomics + genetics	*n* = 2	^44,46^
EHR+ Microbiome	*n* = 1	^36^
EHR+ Fundus images	*n* = 2	^33,43^
EHR+ CT	*n* = 2	^34,56^

Regarding data resources, the included studies used datasets from both private and public sources. Private datasets originate from hospitals and clinics, typically inaccessible to the public. On the other hand, public datasets are made available to the general public and are often collected by government agencies or research institutions to be used for research purposes. Of the 40 studies, 22 used publicly accessible datasets^20,21,24,28,29,31,32,36,38–43,45,47–53^, while 18 studies relied on private data sources^11,18,19,22,23,25–27,30,33–35,37,44,46,54–56^. It is worth noting that almost all of the public datasets are not freely accessible due to containing sensitive personal health information. Access to the datasets is granted on a case-by-case basis and requires approval from the study’s committee. Therefore, researchers might need special permissions to access them, and some datasets may require payment of access fees. The most frequently mentioned public datasets in the studies were the Tehran Lipid and Glucose Study (TLGS), Canadian Primary Care Sentinel Surveillance Network (CPCSSN), and San Antonio Heart Study (SAHS), each used in three studies. Other datasets were mentioned in only one study. Supplementary Table 4 elaborates on these datasets, while Table 3 summarizes the predominant datasets from the included studies.

**Table 3:** The most commonly used public datasets.

Dataset name	Description	Population	Data link	Study reference
SAHS	It is a longitudinal epidemiological investigation that seeks to explore the incidence of T2DM and cardiovascular disease in the population of San Antonio, involving both Mexican Americans and non-Hispanic whites.	USA	SAHS Website	^21,31,38^
CPCSSN	It is a primary care electronic medical record surveillance system that collects data from participating primary care clinics in Canada for various health research purposes, including the development and evaluation of chronic disease management programs, monitoring disease trends, and improving primary care services	Canada	CPCSSN Website	^49–51^
TLGS	It is a large-scale, longitudinal, population-based study conducted in Tehran, Iran. The study was initiated in 1999 to assess the prevalence and incidence of non-communicable diseases and their risk factors among Iranian adults.	Iran	TLGS Website	^39–41^

Beyond data types and sources, the study populations showcased a wide geographical and demographic diversity. Participants originated from various regions, including the Han ethnicity in China^46^, the Finnish population^36^, non-Hispanic Caucasians in California^48^, and native Arabs in Kuwait^22^. The gender distribution in these studies also varied, with some populations being male-dominated^32,36^, others having a female majority^38,48^, and some exhibiting balanced gender representation^24,33^. However, several studies did not provide gender details^34,43,49–51,54,55^. Supplementary Table 5 offers a detailed overview of the country, gender, and ethnicity representation across studies.

In this scoping review, the studies showcased a wide variation in sample sizes used to develop T2DM risk prediction models, with sizes ranging from as few as 244 patients^48^ to an extensive dataset of up to 1,893,901 patients^30^. Approximately half of these studies provided rationales or calculations for their chosen sample sizes. Most of them derived their sample size based on available data and specific eligibility criteria^21,22,24,25,27,31,35,36,39,42–44,48,52,53,55^, an approach commonly found in studies that used existing datasets. Two studies highlighted constraints on size due to data availability^19,46^, while the availability of fasting serum samples constrained another one^24^. Additionally, one study^56^ adopted a case–cohort design, in which the sample size was calculated using a 1:4 case-to-control ratio. This ratio was considered favorable for maintaining relative efficiency and helped ensure an adequate representation of cases and controls. However, the remaining studies did not specify a rationale or methodology for determining their sample sizes.

In our review, the class distribution in the datasets used in the majority of the included studies was imbalanced (*n* = 34)^11,18,20–23,25–27,29,31–33,35–42,44,45,47,49–51,53–56^. Such imbalance can introduce model bias. To mitigate this issue, 14 of the studies adopted various techniques. Six utilized the Synthetic Minority Oversampling Technique (SMOTE) to generate synthetic samples for the less-represented (positive) class^21,25,28,37,39,40^, while three opted for downsampling^25,49,55^. Two studies partitioned their datasets ≥ 100 times into training and testing balanced datasets to address the class imbalance^11,43^, and two applied class weighting during training^19,27^. For three studies, methods to address class imbalance were unclear and only described addressing class imbalance using 10-fold cross-validation^24^, jackknife or “leave one out” procedure^21^, and stratified random sampling^41^.

Missing data is a common challenge in research studies and can significantly impact the results and conclusions of the study. Despite its importance, only 20 studies in this scoping review reported how they handled missing data. The most common method was the removal of rows containing missing data before training the AI model, an approach utilized by 12 of these studies^19–23,26,36,38,51,53,56^. While straightforward, this method can restrict the data used for model development and potentially introduce bias if the remaining sample is not representative^4,5^. Eight studies used imputation techniques to fill in the missing data. These included K-nearest neighbor (KNN) imputation^29^, Gaussian imputation^52^, classification, and regression tree (CART) imputation for continuous variables^39^, a nonparametric imputation method based on random forest^37^, and imputation using the arithmetical mean of the corresponding variable^50^. Only one study^55^ compared various methods, such as data removal and mean, median, and mode imputation. Another study^30^ did not detail its approach to missing data.

### Modeling approaches

The type and number of data modalities used to train a prediction model can significantly affect the model’s performance and impact the model’s reliability and prediction outcomes. With this in mind, we categorized the AI models developed in the reviewed studies into unimodal and multimodal categories. Unimodal models used a single type of data as input, whereas multimodal models incorporated multiple data sources as input.

#### Unimodal predictive models

In this scoping review, 30 studies used unimodal models for T2DM risk prediction, which accounted for the majority of the included studies^11,18–23,25–32,37–42,45,49–56^. A summary of their characteristics can be found in Supplementary Table 6. Various AI algorithms were utilized to develop these predictive models, with classical ML being the most frequently employed compared to DL models. The classical ML models employed were very diverse. Among them, decision trees (DT) and their variants, such as CART, quick, unbiased efficient statistical tree (QUEST), commercial version (C5.0), and DT using the CHAID method, were the most widely used, with ten studies implementing them. Moreover, linear regression (LR) was used in ten studies. Random forest (RF) was used in nine studies, while support vector machines (SVM) were implemented in eight studies. Naïve Bays (NB) classifiers were implemented in five studies, while KNN and extreme gradient boosting (XGBoost) were utilized in four studies each. Ensemble learning, which involves combining the predictions of multiple models to improve the overall accuracy of the prediction, was used in four studies employing different forms of voting, such as soft voting and weighted voting. Hidden Markov models (HMM) were used in three studies, while gradient boosting machine (GBM) and linear and quadratic discriminant analysis (LDA and QDA) classifiers were used in two studies each. The least used algorithms were K-means, AdaBoost, Cox regression, and multiple instances learning boosting (MIL-Boost) with one study each. Regarding DL algorithms, feed-forward neural networks (FFNN) were used in three studies, followed by long short-term memory (LSTM) and probabilistic neural network (PNN) in one study each. Table 4 presents the distribution of unimodal AI models across different studies.

**Table 4:** Distribution of unimodal AI models in studies.

Unimodal AI models	Study references
Classical ML models	
DT	^19,25,26,39–42,52–54^
LR	^11,20,22,23,25–27,37,52,53,55^
RF	^11,18,20,25,26,37,52,53,55^
SVM	^19,22,37,49–52,55^
NB	^25,40,50,52,53^
KNN	^19,22,23,37^
Ensemble models	^25,50,53,55^
XGBoost	^26,30,32,55^
HMM	^21,31,61^
GBM	^20,37^
LDA and QDA	^19,23^
K-means	^56^
MIL-Boost	^29^
AdaBoost	^50^
DL models	
FFNN	^20,28,37^
LSTM	^45^
PNN	^40^

Around half of the unimodal studies compared several ML algorithms and then selected the best-performing one for prediction. In studies that compared two or more algorithms, DT was found to have the best performance in five studies^19,39,41,42,54^. Meanwhile, ensemble models performed best in four studies^25,50,53,55^. RF^11,20,52^, XGBoost^26,30,32^, and HMM^21,31,38^ each had the best performance in the three studies. The performance of the algorithms was measured using the area under the curve (AUC) metric in most of the studies (*n* = 23). The best-selected models in the unimodal studies had AUC values ranging from 0.74 to 0.92, with three studies having values below 0.74 and only one exceeding 0.92. However, it is crucial to note that these results are based on individual studies and are not directly comparable due to the use of different datasets and risk predictors, evaluation metrics, and follow-up periods across studies.

All unimodal predictive models used EHR data as input except one study that used imaging^56^. The EHR risk factors and biomarkers used as inputs by the included studies can be broadly categorized as (1) sociodemographic and FHD; (2) lifestyle; (3) anthropometric measures of body size and composition; (4) glycemic traits that include measures of glucose control; (5) blood lipid and blood pressure factors that include measures of cholesterol, triglycerides, and blood pressure; (6) inflammatory biomarkers including measures of inflammation, such as C-reactive protein-to-albumin ratio (CAR); (7) other biomarkers including measures of liver function, such as liver enzyme levels, or measures of adiposity, such as circulating adiponectin levels; (8) medications and disease history. Supplementary Table 6 includes the different EHR features among studies. Only one study^56^ in this category used imaging data represented in CT scans to investigate the relationship between different fat distribution patterns and the risk of developing T2DM.

Overall, the results showed that unimodal predictive models had moderate to high performance in predicting T2DM risk, with an average AUC of 0.81. However, it is essential to note that it is difficult to determine exactly which AI model is the best, as the type and combination of input risk predictors can significantly influence the model’s performance. For example, the XGBoost algorithm was used in three studies^26,30,32^, and the reported AUC was 0.91, 0.83, and 0.67 for each study, respectively. Each of these studies used different risk predictors, causing the algorithm to excel with certain combinations and underperform with others. Additionally, the results show that the prediction horizon also impacts the performance of the same model. In studies that carried out predictions over varying follow-up periods, we observed that the model’s discriminatory ability tends to decrease as the prediction horizon increases^20,22,45,52^.

#### Multimodal predictive models

Surprisingly, multimodal predictive models were less common in the studies included in this review. A total of ten studies used multimodal predictive models^24,33–36,43,44,46–48^, as shown in Supplementary Table 7. These models employed various fusion strategies, including early fusion and joint fusion, as highlighted in the studies by Huang et al.^57^ and Mohsen et al.^58^.

#### Early fusion

In early fusion, the various data modalities are combined at the data level before feeding them into the AI model. This approach allows the model to take advantage of the complementary information provided by the different data sources. In this review, seven studies adopted an early fusion strategy, integrating various data types, including EHR and multi-omics^24,35,36,44,46–48^. Specifically, two studies combined genetics and EHR features^35,47^, while another two fused metabolomics with EHR^24,48^. Additionally, two studies integrated genomics, metabolomics, and clinical data^44,46^, and one more study integrated microbiome data with EHR^36^.

Five of these studies applied feature selection techniques to eliminate extraneous and redundant features to address the issue of high dimensionality before combining the different modalities^35,44,46–48^. These techniques included Cox proportional hazards (CPH)^35^, J48 DT^48^, random forest feature selection^46^, Boruta algorithm^44^, and L1 penalized logistic regression^47^. The selected features from the various modalities were then concatenated and fed as inputs into a range of AI algorithms for T2DM risk prediction. Classical ML algorithms were the most often used, with RF being the most popular (employed in four studies)^35,36,44,46^. Other algorithms, including GBM^35^, NB^48^, regularized least squares^24^, and J48 DT^48^, were each utilized in one study. Deep neural networks (DNN) were also featured in two studies^35,47^.

Two studies investigated the value of multiple genetic variants in T2DM prediction^35,47^. Both of these studies used these genetic variants in combination with classical risk factors to improve the performance of their AI prediction models. In one study^35^, the findings indicated that prediction by clinical risk factors was significantly enhanced when genetic information was added. Another study by Kim et al.^47^ compared the performance of LR and DNN models by using varying numbers of SNPs. Specifically, when only 96 or 214 SNPs were utilized, both the LR and DNN models demonstrated limited discriminative ability, and they did not surpass the performance of a clinical model built on classical risk factors. However, when more SNPs were included (399 and 678), both models achieved a higher AUC than the clinical-only model. Combining both clinical factors with SNPs significantly enhanced the AUC of the DNN models. Overall, the results of this study suggest that combining genetic data with classical risk factors may improve the performance of AI prediction models for T2DM prognosis, especially when a more extensive set of genetic variants is incorporated. These findings highlight the potential value of incorporating genetic information into T2DM prediction models.

Two studies^24,48^ explored the use of metabolomic data as potential risk predictors for T2DM. The first study^24^ evaluated the entire metabolome’s predictive ability for T2DM and found an AUC of 0.77, compared to a model using only clinical risk factors with an AUC of 0.68. Interestingly, even when limited to a subset of metabolite signatures, the AUC was 0.75, still surpassing the clinical-only model. Combining the panel of selected metabolite signatures with clinical variables achieved the highest predictive performance, resulting in an improved AUC of 0.78. This combination outperformed both the clinical and metabolite-only models, demonstrating the significance of integrating both data types to enhance prediction accuracy. Another study^48^ identified 21 metabolites that significantly differed between non-T2DM and T2DM patients, achieving an AUC of 0.77. However, when this metabolite data was combined with glucose risk factors, the resulting AUC was 0.75. These findings suggest that, in this particular study, metabolomic markers on their own were more effective in predicting T2DM. In conclusion, the included studies underscore the efficacy of metabolomic biomarkers in predicting T2DM.

In two studies^44,46^, the fusion of genomic and metabolomic data with classical risk factors improved the performance of AI prediction models for T2DM. One study^44^ indicated that integrating genomic, metabolomic, and clinical data significantly improved the model performance, achieving an AUC of 0.884. This surpassed both the model integrating genetic data with classical risk factors (AUC of 0.876) and the one relying solely on classical risk factors (AUC of 0.84). Similarly, another study^46^ revealed that a model combining genomic, metabolomic, and clinical risk factors was superior in predicting T2DM, yielding an AUC of 0.96 in comparison to a genomics-only model (AUC of 0.586) and a clinical-only model (AUC of 0.798). These findings suggest that integrating genomic, metabolomic, and clinical predictors has consistently improved the prediction models.

Oliver et al.^36^ conducted the first longitudinal study to assess the gut microbiome’s role as a predictive factor for various parameters associated with T2DM. Their findings revealed that the microbiome, in combination with conventional risk factors, could effectively predict various metabolic outcomes. The authors concluded that using the microbiome in personalized medicine is promising. However, the true potential of the gut microbiome for predicting T2DM remains unknown.

Six of the ten studies employing early fusion compared the performance of fusion models with single-modality models to assess the efficacy of multimodal models^24,35,36,44,46,47^. Five studies found that fusion models performed better than their unimodal counterparts^24,35,44,46,47^. The average AUC value of early fusion models was 0.89, indicating the potential of early fusion multimodal models in improving T2DM prediction.

#### Joint fusion

In this scoping review, three studies employed a joint fusion approach for combining multiple data modalities to develop T2DM predictive models^33,34,43^. These studies fused EHR metadata with different types of medical imaging, such as CT and retina scans, as shown in Supplementary Table 7. They used DL models to extract imaging features and jointly learn multimodal feature representation for T2DM prediction.

One study by Zhang et al.^33^ explored the capability of an AI model to predict the future risk of T2DM in individuals using fundus images and clinical data. The authors proposed a deep multimodal framework to effectively capture complementary features from image and non-image modalities to predict T2DM five years before disease onset. They used deep convolutional neural networks (CNNs), specifically a residual network (ResNet50) architecture, to convert the fundus image data into a feature vector fusible with other metadata. Then, the image feature vector derived from the CNN model was concatenated with the clinical features of the same patient and fed into a multilayer perceptron for joint learning and prediction. Their results revealed that the fusion of fundus images and clinical data considerably enhanced the model’s performance, achieving an AUC of 0.85, compared to AUCs of 0.82 and 0.76 for the fundus-only and clinical-only models, respectively. Drawing from these results, the authors concluded that fusion models of fundus images and clinical data could be used to automate the prediction of T2DM risk in healthy individuals.

Similarly, a study by Yun et al.^43^ also investigated retinal scans with additional traditional risk factors for T2DM screening and prediction using the ResNet18 model. They found a similar trend, where the multimodal approach outperformed the unimodal counterparts. Specifically, the fusion model achieved an AUC of 0.84, considerably exceeding the clinical-only and fundus-only models, with AUCs of 0.81 and 0.73, respectively. Another study^34^ explored the feasibility of integrating CT images with clinical data to develop a 1-year risk prediction model for T2DM. Pancreatic CT images were processed to extract body composition features, such as abdominal visceral fat volume, subcutaneous fat volume, and pancreas volume, using CNNs. To develop a clinical-image multimodal risk prediction model, the authors combined these imaging features with clinical data and input them into fully connected layers. Their results demonstrated notable improvements in the model performance upon the fusion of the two data sources (AUC = 0.89) compared to the clinical-only model (AUC = 0.82) and the imaging-only model (AUC = 0.85).

The three studies^33,34,43^ compared the performance of their joint fusion multimodal models with single modality models, whether clinical-only or imaging-only. All of them showed superior performance with fusion compared to single-modality models. The average AUC value for the joint fusion models was 0.86, ranging from 0.84 to 0.89. These findings highlight the potential of joint fusion models to enhance T2DM risk prediction.

### Evaluation and performance metrics

The next crucial step following model development is performance evaluation. This can be done in two ways: (1) internal validation, which involves evaluating the model’s performance on the same dataset used for training, such as split sampling or cross-validation techniques, and (2) external validation, which involves using an entirely different dataset. In this review, almost all studies (*n* = 39) employed internal validation.

When validating a prediction model’s performance internally, the holdout method is not deemed optimal because it reduces the available sample size^59,60^. However, it emerged as the most commonly employed method in the reviewed studies (*n* = 18)^11,18,26,27,31–33,35–41,45,47,53,54^. Although cross-validation techniques are preferred as they utilize the entire data for both model development and validation^59,60^, K-fold cross-validation was adopted in 17 studies^19,23–25,28,29,42,44,46,48–51,55^. This approach partitions data into k equally sized folds, training and evaluating the model k times, each using a different fold as the test set. Three other studies^20,30,43^ employed the train-valid-test method, dividing the dataset into training, validation, and testing subsets. Only one study utilized the leave-one-out cross-validation method^21^, while another study^56^ did not mention its validation approach.

A smaller proportion of the included studies (*n* = 5) conducted external validation to assess their predictive model’s generalizability^24,32,33,43,56^. These studies utilized diverse cohorts spanning several countries: France^24^, Germany^56^, China^33^, Australia^43^, and Japan^32^. These cohorts’ geographical locations differed from their respective development datasets, reflecting efforts to validate the predictive models across various contexts. Supplementary Table 8 details the specifics of each cohort, including sample size, country, gender distribution (where available), and other pertinent information.

The evaluation metrics used in the reviewed studies were highly diverse. The majority of studies utilized discrimination metrics, particularly the AUC (*n* = 32). Standard classification measures were also reported in almost half of the studies, including accuracy, specificity, precision, sensitivity, and F1 score. Other metrics, such as the Youden index, net reclassification improvement (NRI), integrated discrimination improvement (IDI), root mean squared error (RMSE), Jaccard similarity, positive predictive value (PPV), negative predictive value (NPV), Cohen’s Kappa, geometric mean (G-Mean), and Matthews Correlation Coefficient (MCC) were also utilized. While calibrating AI models is crucial for predictive performance assessment, only a few studies (five in total) evaluated their models’ calibration using measures such as the Brier score, calibration plot, and Hosmer-Lemeshow test. The distribution of the most common reported performance measures used across the included studies is presented in Table 5.

**Table 5:** The distribution of evaluation metrics in the included studies.

Performance measures	Number of studies	References
AUC	*n* = 32	^11,18–35,37–39,43–48,50,52–54^
Accuracy	*n* = 15	^11,19,25,26,28,29,34,40–42,48–51,55^
Specificity	*n* = 15	^11,19,25,26,28,39–41,45,46,49–53^
Sensitivity	*n* = 20	^11,19,25,26,28,29,33,34,39–41,45,46,48–53,55^
Precision	*n* = 7	^25,29,34,40,41,48,55^
F1 score	*n* = 8	^26,29,39,44,48,50,53,55^
Positive predictive value	*n* = 5	^19,39,45,53,55^
Negative predictive value	*n* = 5	^19,39,45,53,55^
Net reclassification improvement (NRI)	*n* = 4	^35,43,44,47^
Integrated discrimination improvement (IDI)	*n* = 3	^24,44,47^
Brier score	*n* = 2	^35,44^
calibration plot	*n* = 2	^30,32^
Hosmer–Lemeshow test	*n* = 1	^45^

#### Performance comparison of unimodal and multimodal T2DM predictive models

The included studies showed promising performance for AI models for T2DM risk prediction. Unimodal models showed widely varied performance with an average AUC of 0.81. In comparison, multimodal models displayed a notably superior performance compared to their unimodal counterparts, achieving an average AUC of 0.88. However, it is crucial to note that direct comparisons across these studies should be taken with caution due to variations in their datasets, evaluation metrics, and prediction horizons.

The improved performance of multimodal models can be attributed to the augmented information available through integrating multiple data modalities, providing a comprehensive view of an individual’s health status. However, they also have some limitations and challenges. They often face scalability issues, and the data concatenation process can be time-consuming^61^. The inherent differences in data types, distributions, and scales across modalities can pose difficulties in effectively integrating the data and building prediction models. Additionally, they often require significant computational resources, which can be challenging when working with large datasets. Understanding the relationships between various modalities within the multimodal frameworks and discerning how each modality contributes to the overall prediction remains challenging. Overall, multimodal models, despite their advantages, have complexities that must be addressed for effective clinical integration.

### Interpretation and risk predictors

Feature ranking and explainability are essential aspects of any predictive model, as they can provide insights into which factors are most important in driving the prediction. In the context of T2DM, this can clarify which risk factors and biomarkers are most influential in determining disease progression. This can help healthcare professionals identify areas to target for intervention and prevention. Moreover, interpretable models can enhance the trust and acceptance of AI-based predictive tools among healthcare professionals and patients.

In this scoping review, nearly half of the included studies reported on feature ranking and explainability techniques. Some studies utilized permutation feature importance^19,29,51,55^, while others relied on the built-in feature importance functions of algorithms, such as decision trees^39,41,42^, XGBoost^26,32^, RF^11,46^, and HMM^21^. Three studies employed LR for feature importance^27,37,38^, and two studies ranked the relative importance of risk predictors based on their contribution to variance^20,45^. Four studies elucidated risk predictors using Shapley plots^26,29,30,32^. In a study that used DL^33^, the model was interpreted using the integrated gradient algorithm to pinpoint the most critical areas in the image.

#### Risk factors and biomarkers

Nearly half of the studies (*n* = 21) reported the final risk predictors identified by their AI models. Table 6 summarizes these predictors across the studies included in this scoping review.

**Table 6:** List of reported biomarkers and risk factors.

Data type	Biomarker/risk factor category	Biomarker/risk factor	Number of studies	Study References
EHR	Anthropometric measures	BMI	*n* = 13	^11,20–22,26,31,32,37,38,41,42,45,55^
		Waist circumference (WC)	*n* = 3	^11,26,42^
	Biochemical markers	fasting plasma glucose (FPG)	*n* = 11	^11,21,26,31,32,38,39,41,42,45,55^
		glycated hemoglobin (HbA1c)	*n* = 5	^11,27,31,38,55^
		triglycerides (TG)	*n* = 11	^11,21,26,27,29,31,38,41,42,45,55^
		high-density lipoprotein (HDL)	*n* = 5	^11,21,29,32,38^
		low-density lipoprotein (LDL)	*n* = 2	^32,38^
		alanine transaminase (ALT)	*n* = 5	^26,27,29,32,45^
		aspartate transaminase (AST)	*n* = 2	^29,45^
		Total cholesterol (TC)	*n* = 3	^26,27,38^
		Gamma-glutamyl transferase (GGT)	*n* = 2	^45,55^
	Sociodemographic data	age	*n* = 9	^19–22,31,32,38,42,55^
		sex	*n* = 4	^22,42,45,55^
	Medical history	family history of diabetes (FHD)	*n* = 5	^20,22,37,41,55^
	Lifestyle factors	alcohol intake	*n* = 3	^20,45,55^
	Blood pressure	Blood pressure	*n* = 6	^20–22,27,38,45^
	Other predictors	Other risk predictors^a^	*n* = 1	
Multi-omics	Metabolomic biomarkers	novel markers (*α*-tocopherol, [Hyp3]-BK, X-12063, and X-13435) and known markers (glucose, mannose, and *α*-HB)	*n* = 1	^24^
		PC ae C40:5 and SM (OH) C14:1	*n* = 1	^48^
		ribofavin, cnidioside A, 2-methoxy-5-(1H-1, 2, 4-triazol5-yl)- 4-(trifuoromethyl) pyridine, 7-methylxanthine, and mestranol	*n* = 1	^46^
Imaging	Retinal biomarkers	Retinal biomarkers such as vascular tortuosity, venous dilatation, retinal hemorrhage, and cotton wool spots	*n* = 1	^33^

^a^Other risk predictors appearing only once in the final model: 2-hour postprandial plasma glucose (2h-PCPG)^24^, waist-to-height ratio (WHtR)^24^, family history of hypertension^22^, smoking^55^, physical activity^55^, income^20^, health insurance^20^, occupation^41^, chronic liver disease^27^, dyslipidemia^20^, hypertension^20–22,27^, cardiovascular disease^20^, obstructive sleep apnea^27^, hypersomnia with sleep apnea^27^, hyperlipidemia^27^, anemia^27^, impaired fasting glucose^27^, acute bronchitis^27^, abnormal blood chemistry^27^, medications (Metformin^27^, antiarthritics^27^, nonsteroidal anti-inflammatory drugs^27^), serum albumin^22^, serum uric acid^55^, serum aldosterone^11^, serum leptin^11^, hematocrit^22^, urea^22^, health insurance^20^, blood glucose level (BGL)^37^, left ventricular mass^11^, Mean Arterial Pressure (MAP)^41^, sodium^22^, Inflammatory Markers (high Sensitivity C-Reactive Protein (hs-CRP)^19^, log(hs-CRP)^19^, fibrinogen^19^), Homeostatic Model Assessment of Insulin Resistance (HOMA-IR)^19^, Change in Glucose Level From 120 to 60 Minutes After a Meal (Δ*G*120 − 60)^51^, Change in Glucose Level From 30 to 0 Minutes After a Meal (Δ*G*30 − 0)^51^, Area Under the Glucose Curve From 0 to 120 Minutes After a Meal (AuG0-120)^51^.

The reported risk factors and biomarkers for T2DM progression showed variation across studies. EHR-based predictors emerged frequently, encompassing anthropometric measures, glycemic traits, blood lipids, sociodemographic data, and liver enzymes. The risk predictors derived from EHRs align well with established literature and are recognized for their biological relevance to the disease. Among these, BMI, FPG, TG, and age stood out as the most frequently reported predictors. Additionally, liver enzyme biomarkers, specifically ALT and AST, were highlighted in five and three studies, respectively. Yet, certain biochemical markers associated with T2DM risk, such as inflammatory biomarkers (hs-CRP and fibrinogen), plasma adiponectin, leptin, albumin, and aldosterone, were infrequently explored in the context of disease prediction.

Three studies reported metabolomic biomarkers, whereas one study reported retina scan-based biomarkers. In terms of metabolomic biomarkers, one study^24^ identified novel markers associated with T2DM progression, such as *α*-tocopherol, [Hyp3]-BK, X-12063, and X-13435, as well as known markers like glucose and mannose. Another study^46^ identified five newly discovered metabolic markers, including iboflavin, cnidioside A, 2-methoxy-5-(1H-1, 2, 4-triazol-5-yl)-4-(trifluoromethyl) pyridine, 7-methylxanthine, and mestranol. A study^48^ provided insights into the etiology of the transition to T2DM in women who previously had gestational diabetes mellitus, revealing two predictive metabolites for incident T2DM: Phosphatidylcholine acyl-alkyl C40:5 (PC ae C40:5) and Hydroxysphingomyeline C14:1 (SM (OH) C14:1). In the realm of imaging-based biomarkers, a study^33^ pinpointed retinal markers associated with T2DM development. These markers, such as vascular tortuosity, venous dilatation, retinal hemorrhage, and cotton wool spots, are frequently employed by ophthalmologists for diagnosing retinal diseases.

Some multimodal models have combined polygenic risk scores with metabolomic markers^24,48^. However, they often do not detail the predictors or interaction and contribution of the various modalities in the final prediction, making it challenging to understand how these multimodal models make predictions and decisions, which can be a barrier to their adoption in clinical practice^62^. There’s a need to investigate shifts in feature importance across both unimodal and multimodal contexts, as it can provide insights into the impact of the multimodal setting.

In interpreting AI-based disease risk prediction studies, it is important to distinguish between causation and correlation. Although AI models can identify features that are strongly correlated with disease outcomes, these correlations do not necessarily infer causation. To critically evaluate the interpretation of the findings in our scoping review, we closely examined how studies elucidated the relationship between features and T2DM. We found that all studies emphasized the predictive nature of the identified associations when interpreting their models and refrained from making causal inferences based on their findings.

### Reproducibility and reporting standards

Transparency and reproducibility are fundamental pillars of robust scientific research. In the context of AI-based predictive modeling, this involves adhering to established reporting guidelines and making the implementation code publicly accessible. In this review’s studies, adherence to established reporting guidelines was not frequently mentioned. These guidelines aim to enhance research transparency and offer a comprehensive understanding of the methods employed and the results. Out of the 40 studies, only three^30,32,42^ explicitly acknowledged their adherence to the Transparent Reporting of a multivariable prediction model for Individual Prognosis or Diagnosis (TRIPOD) reporting guidelines^63^. This highlights the need for more transparent and rigorous reporting, especially given the potential for such models to impact healthcare outcomes significantly. Additionally, making implementation codes publicly accessible is crucial for reproducibility. Of all the studies analyzed, only four^29,36,38,44^ made their code publicly available, highlighting the need for improved reproducibility in future research.

## Discussion

This scoping review has comprehensively analyzed the current state of AI-based models for T2DM risk prediction in the published literature. This section summarizes the key findings and outlines potential future directions for research in this area.

A total of 40 studies were included, and the results showed promising performance for AI models for T2DM risk prediction. Different data modalities and modeling techniques were used to develop these prediction models. EHR data was the most common data type used in the included studies. This data is often used alone or in combination with other modalities, such as multi-omics and imaging data. Multi-omics data, including genomics and metabolomics, were the second most used data modality, while imaging data such as CT and retinal scans were the least used data.

The majority of studies in our review used unimodal AI models to predict the risk of T2DM. These studies used different AI algorithms to develop predictive models, with classical ML models being the most widely used, such as tree-type (DT and RF), SVM, KNN, and ensemble learning models. Unimodal models have shown moderate to high performance with an average AUC of 0.81. However, it’s crucial to note that determining the best-performing model is challenging. This is because the type and combination of input risk predictors can significantly influence the performance. For example, the XGBoost algorithm was used in three unimodal studies^26,30,32^, yielding AUC values of 0.91, 0.83, and 0.67, respectively. Each of these studies utilized distinct datasets with varying sample sizes and combinations of risk predictors. This variability likely influenced the algorithm’s performance, leading it to excel with certain combinations but not with others. Moreover, the duration of the prediction horizon also significantly impacts the prediction performance of the same model. In studies that performed prediction over different time periods^20,22,45,52^, we observed that the discriminatory power decreased as the prediction horizon increased. Unimodal models, while useful, may not capture the complexity of T2DM risk prediction as the individual’s state is characterized by a spectrum of data modalities, ranging from EHR and multi-omics to imaging. Such single-modality models neglect the broader clinical context, which inevitably diminishes their potential. On the other hand, multimodal models have the advantage of incorporating multiple data sources, providing a more holistic view of the individual and potentially improving the prediction performance.

In this scoping review, a smaller proportion of the included studies employed multimodal AI models. In these models, the most frequently used data combinations were multi-omics integrated with EHR, as well as imaging data paired with EHR metadata. Notably, no study within our review integrated the three data sources of multi-omics, imaging, and EHR into one multimodal predictive model. These multimodal studies predominantly used two fusion strategies: early fusion and joint fusion. Early fusion was the dominant approach for multimodal learning, commonly combining multi-omics with EHR data. Conversely, joint fusion was used less frequently, mainly integrating imaging with EHR data. Regarding predictive performance, our scoping review found that multimodal models generally outperformed unimodal ones, with an average AUC value of 0.88. Most of the multimodal studies compared their results with their unimodal counterparts, demonstrating improved performance when leveraging multimodal data^24,33–35,43,44,46,47^. This finding aligns with previous reviews on cancer research^62^ and cardiovascular disease care^64^. A primary advantage of multimodal AI models is their ability to identify complex interactions between various data modalities, which may not be apparent when using a single data modality. Therefore, they can result in more accurate risk predictions, paving the way for personalized prevention and management strategies to be developed for individuals at high risk for T2DM.

However, developing multimodal models comes with challenges, such as the time-consuming nature of their development, data concatenation, and lower scalability^61^. Their complex nature, often merging multiple data sources, also complicates understanding the interactions among modalities and the rationale behind predictions. Such interpretability issues could impede their clinical adoption and represent a challenge for clinicians and researchers who need to understand the underlying mechanisms and reasoning behind the models’ predictions to use them in clinical practice. Given the scarcity of multimodal AI models for T2DM, further research is needed to investigate their use and to identify the best data fusion strategies. Additionally, it is crucial to focus on interpretability and explainability during their development to facilitate their integration into clinical workflows.

Despite the promising results of AI models for T2DM risk prediction, it is worth noting that the studies in this scoping review showed considerable variation in the quality and comprehensiveness of performance reporting. Adequate information on the various dimensions of predictive performance, such as discrimination and calibration, is crucial in determining the effectiveness of a prediction model. However, only a limited number of studies reported calibration measures, and many reported only a single dimension of performance, such as the AUC or classification measures. Uncalibrated models may have limited applicability in practical, real-world situations^65^. The lack of detailed performance reporting in the included studies presents a challenge when determining the generalizability and practicality of these AI models in real-world settings. Consequently, we recommend that future studies emphasize the comprehensive reporting of their models’ performance, including discrimination, calibration, and classification metrics. Moreover, we recommend standardizing evaluation metrics across studies to enable more consistent and comprehensive comparative assessments. This can be achieved by adopting uniform evaluation metrics across studies to streamline comparisons. We suggest incorporating metrics such as AUC, sensitivity, specificity, precision, F1-score, and calibration metrics to holistically evaluate model performance. Furthermore, algorithmic fairness should not be overlooked. Evaluating the model’s performance across diverse demographic groups using fairness metrics, such as demographic parity^66^ and equal opportunity^67^, is essential. Such measures will enhance our understanding of the validity and applicability of AI predictive models.

Despite the advancements in the development of AI-based prediction models for T2DM prognosis, this scoping review identifies some barriers that hinder the progress of knowledge and the clinical utility of these models. A predominant barrier is the reliance of most of the included studies on the hold-out internal validation approach. The performance estimate using this approach can highly depend on which data points end up in the training set and which end up in the validation set. This can lead to high variance in the performance estimate, which can make it difficult to assess the true performance of the model. Moreover, hold-out validation presents a concern as it reduces the sample size available for model development and may not utilize the data effectively^59,60^. Therefore, we recommend that future studies place greater emphasis on the method of validation of the developed predictive models. Techniques such as cross-validation or bootstrapping can be used for internal validation.

Another barrier is the limited external validation conducted, with only five studies performing it. This limitation raises concerns about the generalizability of these models, which in turn restricts their practical implementation. This finding aligns with previous reviews that noted a lack of external validation for prediction models^68^. There is growing evidence that many areas of scientific research are experiencing a replicability crisis, including precision psychiatry^69^, genetic behavior research^70^, and cancer research^71^. Therefore, we recommend conducting external validation and testing of AI models in different settings and populations to establish a robust foundation for their clinical implementation and enhance their potential to guide T2DM prevention strategies.

Machine learning is inherently iterative; thus, the optimal predictive model could be generated by comparing a combination of algorithms^16^. However, the included studies did not frequently adopt such extensive modeling. For instance, only half of the studies compared multiple algorithms and selected the best one, while the others developed and tested one algorithm. Unsupervised ML, which can reveal the inherent structures and patterns within multidimensional data^72^, was also rarely utilized. Therefore, we recommend that future research consider testing multiple AI models to identify the most precise one.

Interpretability and explainability methods, such as permutation importance or Shapley values, can provide insights into which variables significantly influence the predictions. The capability of AI techniques to learn abstract feature representations raises concerns about the possibility of the models relying on fake shortcuts for predictions rather than learning clinically relevant information. This may result in models that cannot generalize effectively when faced with new data or exhibit discriminatory behavior toward specific populations^25,26^. On the other hand, AI models could identify clinically relevant markers, enabling precision medicine and allowing clinicians to personalize prevention strategies and therapies based on patient risk profiles. Unfortunately, many studies in this review did not interpret the predictions made by their model. They relied solely on performance metrics to indicate the high performance of their models. Understanding the reasoning behind a model’s predictions is of utmost importance, especially in a clinical context. The objective of clinical machine learning studies is not only merely prediction but also garnering meaningful insights. Hence, there has been a shift from focusing solely on prediction performance to placing greater emphasis on understanding algorithm dynamics, a notable trend in recent research^73,74^.

Identifying risk predictors for T2DM is vital for disease prevention and guiding targeted interventions for at-risk individuals. However, a mere half of the studies in this review reported the risk predictors identified by their models. Importantly, these studies emphasized the predictive nature of the identified associations, avoiding implying causal relationships based on their findings. Most of them reported traditional risk predictors, such as BMI, blood cholesterol measurements, FPG, age, FHD, and HbA1c, which were consistent with prior research findings. With the advancement of molecular biology and medical imaging, several molecular markers, such as gene expression, metabolomic, and imaging markers, have become potential predictors for T2DM. Few multimodal studies unveiled new metabolomic and imaging biomarkers. However, these studies did not comprehensively report the different biomarkers of the various modalities and did not sufficiently demonstrate the interaction and contribution of the diverse modalities to the final prediction. Therefore, we recommend that future multimodal studies in this field offer a comprehensive understanding of the biomarkers from combined modalities and elucidate the interactions and contributions of these predictors. This approach would enhance the models’ interpretability and facilitate their application in clinical settings.

**Fig. 3:**
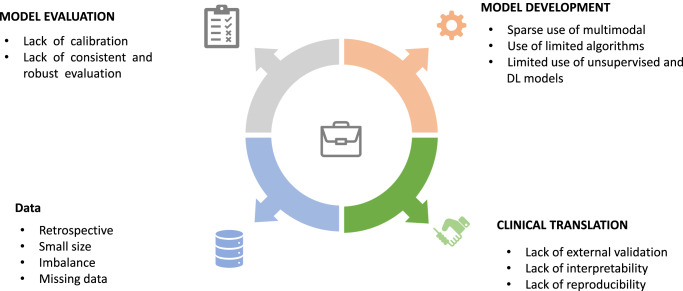
Limitations in AI-based T2DM risk prediction models. The limitations encountered at different stages of AI predictive model construction for T2DM: those associated with the underlying data, the model building and evaluation, and clinical translation.

Additionally, this review highlights several methodological flaws raised in the studies that hinder the implementation of AI in clinical settings and precision medicine. These limitations include small sample sizes, retrospective data, imbalanced samples, and inadequate handling of missing data. Small sample sizes often result in poor model fitting and generalizability, with some studies having as few as 244 participants and fewer than 1000 in six studies. A sizeable proportion of the studies (nearly half) did not justify their chosen sample sizes, a factor that introduces the risk of overfitting, particularly when complex ML models are utilized. Moreover, in instances where sample size justifications were provided, they were primarily based on data availability and specific inclusion criteria. Remarkably, no studies provided justifications for their selected sample sizes in relation to the number of candidate predictors employed during model development. Findings from simulation studies recommend that most ML approaches necessitate over 200 data points associated with the outcome for each candidate predictor to attain stable performance and avoid overly optimistic models^75^. Another methodological flaw is the limited number of investigations based on prospective data, with most models developed retrospectively from research datasets assembled for other purposes. Neglecting sample imbalances often leads to biased models and misleading performance metrics^76^. Additionally, the inadequate handling of missing data can skew the results; therefore, comparing different imputation methods should be part of the reporting process^76^. Figure 3 summarizes the limitations of the included studies in terms of data, model development, evaluation, and clinical translation.

Our review highlights several concerns related to bias and algorithmic fairness within the scope of the studies included. Primarily, the variability of demographic representation across studies is a major point of concern. For instance, some studies showed a pronounced lack of gender balance, focusing exclusively on male^36^ or female participants^46^. Others, however, completely omitted gender information^34,43,54^. The demographic restrictions often centered around specific ethnicities, like the Han ethnicity^46^, or specific age groups, such as middle-aged or older participants^20,22,37,53^. In addition, age representation proved to be challenging, with some studies restricting their scope to specific age groups. For instance, one study centered on individuals 65 years and older^26^, potentially impacting the generalizability of the results. Therefore, this limited diversity might introduce algorithmic bias and undermine the generalizability of the predictive models. Furthermore, our analysis highlighted variations in class balance within the datasets across the studies. Some studies^24,30,46,47^ managed a balance between progressor and non-progressor groups, while others^35,36,45,56^ did not. Using imbalanced datasets can introduce bias into the performance of AI models, as they might perform well on the overrepresented class but poorly on the underrepresented one.

Moreover, most of the studies in our review did not evaluate their algorithms’ performance across different demographic groups, nor did they utilize fairness metrics to assess the disparities in predictions across these groups. The majority of studies reported common evaluation metrics such as AUC, precision, recall, and F1-score, but there was a significant absence of calibration metrics in the evaluation process. The absence of calibration assessments may inadvertently introduce a bias in the model’s predictions, affecting its fairness. Moreover, the identified lack of external validation also raises questions about the fairness of the algorithms. If the training data lack diversity and do not adequately represent the broader population, the derived algorithm might be biased towards the specific demographics included in the training data. This would further limit its applicability and fairness when applied to other demographic groups. Therefore, future research should adopt a comprehensive approach to ensure unbiased and fair algorithm development. Firstly, it is vital to ensure demographic representation in their datasets, which includes gender, ethnicity, and age distribution. Secondly, integrating appropriate evaluation metrics, including calibration metrics and explicit fairness evaluations, is essential for creating models to ensure accurate predictions across various groups. External validation is another crucial aspect. It ensures that models are robust and can generalize beyond the specific datasets used for training, further aiding in detecting and mitigating potential biases. Such a comprehensive approach can drive the development of fair and unbiased AI models, which can help prevent the exacerbation of existing health disparities and promote equitable health outcomes^77,78^.

Of particular concern was the lack of adherence to established reporting standards like the TRIPOD guidelines among the included studies. Such approaches are designed to foster transparency and offer a comprehensive understanding of the methodology and results. Therefore, future studies need to prioritize adherence to such reporting standards in order to enhance research quality and inspire trust in AI models among healthcare practitioners and policymakers.

It is important to note that reproducibility is a key aspect of scientific research, and the development of AI models for T2DM prognosis is no exception. The availability of model code and data is an essential aspect of reproducibility, allowing other researchers to verify and build upon the work independently. In this scoping review, we found that most of the included studies did not report their model’s code or data availability. This lack of reporting of model code and data availability can impede the replication and validation of the models, hindering research progress in this field. Due to privacy regulations, such as the “Health Insurance Portability and Accountability Act” (HIPAA), sharing medical data may not be feasible. However, other measures to promote transparency can be taken, such as requiring authors to provide a summary of their data sample and statistical information about the complete dataset, including the number of data points, key variables, distribution, and class information. A more ideal solution would be to create a synthetic dataset derived from the original data^79–81^. Future studies should emphasize the availability of model codes and data to ensure other researchers can independently verify and replicate their findings. This would improve research reproducibility and facilitate the validation and implementation of the models in real-world settings.

While this scoping review provides valuable insights into the use of AI models for T2DM risk prediction, several inherent limitations should be considered. The literature search was restricted to English-language studies and excluded gray literature, which may result in some studies being omitted. Nevertheless, it is unlikely that the inclusion of additional articles in the review would have significantly impacted the findings. Secondly, the inclusion criteria for this review were narrow, only including studies that specifically evaluated the use of AI models for T2DM risk prediction. As a result, this review does not capture the full spectrum of T2DM research in conjunction with AI. In addition, as this study’s focus was to provide a detailed profile of AI models for T2DM risk prediction, a thorough evaluation of the individual methodological quality of the included studies was not conducted. However, insights were offered on the potential limitations in methodology that may have influenced the results. Because positive results are typically reported disproportionately, publication bias might be another limitation of this review. This bias may result in overestimating the benefits of AI-based models in risk prediction. There is a significant heterogeneity among the studies included in this review in terms of the data sources, study populations, and evaluation metrics, making it difficult to directly compare the results of different studies. Finally, this scoping review only covers the current state of the use of AI models for T2DM prognosis and does not provide a comprehensive evaluation of their potential benefits. Thus, future studies are needed to further evaluate the feasibility, accuracy, and potential benefits of using AI models for prediction.

In conclusion, our study provides a scoping review of AI predictive models in T2DM risk prediction. We observed an increasing trend in the literature toward using both unimodal and multimodal AI models. Our findings suggest that AI models have promising potential in predicting the future development of T2DM. While unimodal models have shown varied performance, multimodal models demonstrated improved performance compared to their unimodal counterparts. However, some challenges and considerations need to be addressed to realize this potential. Additionally, as with any significant medical advancement, there is a need for thorough validation and evaluation through clinical trials and prospective studies to verify the potential benefits claimed by AI models. The role of AI in medicine is not autonomous but rather a partnership between AI models and human expertise that will drive progress in the field. Despite limitations and challenges, it is our responsibility to capitalize on the benefits of AI methods to accelerate the discovery and translation of advances into clinical practice for the benefit of patients and healthcare providers^82^.

## Methods

In conducting this scoping review, we followed the guidelines recommended by the Preferred Reporting Items for Systematic Reviews and Meta-Analyses Extension for Scoping Reviews (PRISMA-ScR)^83^, as detailed in Supplementary Table 1.

### Search strategy

A systematic search was conducted across four databases, including Scopus, PubMed, IEEE Xplore, and Google Scholar, for studies published from January 1, 2000, to September 19, 2022. A systematic search of MEDLINE was not undertaken since these citations were captured in PubMed. Only the first 100 relevant studies from Google Scholar were considered for the review, as search results beyond this number rapidly lost relevance and were not pertinent to the topic of the scoping review. In addition to the database search, reference lists of the included studies were screened to identify additional relevant literature. Search terms were established through literature searches and domain expertise.

In this scoping review, we focused on studies that used AI-based models for T2DM risk prediction using longitudinal datasets. Therefore, our search string was constructed as follows: ((“Artificial Intelligence” OR “machine learning” OR “deep learning”) AND (“prediction” OR “prognosis”) AND (“diabetes” OR “T2DM”) AND (“longitudinal”)). We adapted the string for each database, using various forms of the terms. The complete search strategy can be found in Supplementary Table 2.

### Inclusion and exclusion criteria

The authorship team jointly developed the selection criterion. To be eligible for inclusion in the review, studies had to meet the following criteria: (1) the study used longitudinal data; (2) the primary aim of the study was to use AI/ML algorithms to predict the future development of T2DM (for our context, AI models meant to be classical ML models, DL models, ensemble learning, etc. as mentioned in the search terms listed in Supplementary Table 2); (3) the study was conducted using human subjects; (4) the study used any medical data including imaging, EHR, multi-omics (we did not limit our study to one or two medical data modalities, and we considered studies that fused different data sources of the same type as multimodal. For instance, a study using genomics with metabolomics was considered multimodal under our premise); (5) only original research, peer-reviewed studies, and conference proceedings published in English were included.

We excluded studies that used classical statistical models, such as regression analysis, or those focused on T2DM classification or diagnosis using cross-sectional data. Additionally, studies related to type 1 diabetes, gestational diabetes, or T2DM-related complications were excluded. We also ruled out research that utilized non-human derived data, non-English publications, review articles, conference abstracts, proposals, editorials, commentaries, letters to the editor, preprints, and short letter articles. All papers underwent a two-person verification for inclusion/exclusion from the manuscript.

### Study selection and data extraction

We utilized the Rayyan web-based review management tool^84^ for the screening and study selection process. One reviewer (F.M.) conducted the literature search. After removing duplicates, citations were screened based on their title and abstract to exclude irrelevant studies. Full-text screening was then undertaken to identify the final set of studies that were included for data extraction. The study selection and data extraction processes were conducted by two reviewers (F.M. and H.R.A.). Discrepancies were resolved through discussion, and if consensus could not be reached, a third author (Z.S.) was consulted.

A comprehensive data extraction form was designed to capture essential information from the included studies. This form was tested on five studies to ensure consistent and accurate data extraction. The extracted information included the titles, first author’s name, publication year, publication type, country of the first author’s institution, study’s aim, sample size, study design, follow-up period, participant’s demographics, methods used to ascertain diagnoses of T2DM, and data source (public or private). Additionally, we recorded the number of different data modalities and their categories, such as EHR, EHR/images, or EHR/multi-omics/images. For instance, a study that used clinical measures (structured EHR) and retina images as inputs for the AI was categorized as the “Imaging/EHR” subtype. We also extracted the type of data used in each modality, such as the type of imaging or multi-omics data.

Regarding the modeling techniques utilized in the included studies, we gathered information on the type of AI implemented (such as ML, DL, or a combination of both), the specific algorithm applied, the data fusion methods used for multimodal models, the strategies employed to manage data imbalance and missing data, and the availability of their model-related code. Additionally, we recorded the validation approach (internal or external), the specifics of the internal validation, and the evaluation metrics used. We also extracted information on the interpretability methods that studies used to determine feature importance, as well as any reported risk predictors. See Supplementary Table 3 for a detailed description of the extracted data.

### Data synthesis

Following data extraction, we used a narrative synthesis, aggregating insights from the data extraction form to identify the main themes surrounding AI’s application in T2DM risk prediction. Given the diversity in data modalities, AI methodologies, implementation details, data sources, and evaluation techniques, our analysis spanned multiple dimensions. Initially, we navigated through the study characteristics, focusing on their demographic details, aims, and design methodologies. Next, we summarized the types and sources of data employed across studies. Delving deeper into the technical aspects, we explored AI modeling approaches, ranging from unimodal to multimodal. This exploration aimed to understand the specific AI algorithms used and the fusion strategies adopted to integrate multimodal data. Concurrently, our synthesis delved into model validation methods and the reported performance metrics. We further looked into model interpretation methods and summarized the reported risk predictors. Concluding our synthesis, we assessed the studies regarding code availability and their adherence to established reporting standards.

### Supplementary information

Supplementary Information File

## Data Availability

All data generated during this study is provided as Supplementary materials.

## Code availability

This study did not involve the utilization of any custom code or mathematical algorithm.
