# Supplementary material for: A scoping review of artificial intelligence-based methods for diabetes risk prediction
Source: NPJ Digit Med. 2023 Oct 25;6:197. doi: 10.1038/s41746-023-00933-5 (PMC10600138; doi:10.1038/s41746-023-00933-5)
Supplement: Supplementary file 1 — Supplementary Information File [file 41746_2023_933_MOESM1_ESM.pdf]

Supplementary Table 1: PRISMA-SCR checklist

SECTION	ITEM	PRISMA-ScR CHECKLIST ITEM	Reported in Section
TITLE			
Title	1	Identify the report as a scoping review.	Title page
ABSTRACT			
Structured summary	2	Provide a structured summary that includes (as applicable): background, objectives, methods, results, and conclusions that relate to the review questions and objectives.	Abstract
INTRODUCTION			
Rationale	3	Describe the rationale for the review in the context of what is already known. Explain why the review questions/objectives lend themselves to a scoping review approach.	Introduction
Objectives	4	Provide an explicit statement of the questions and objectives being addressed with reference to their key elements (e.g., population or participants, concepts, and context) or other relevant key elements used to conceptualize the review questions and/or objectives.	Introduction
METHODS			
Protocol and registration	5	Indicate whether a review protocol exists; state if and where it can be accessed (e.g., a Web address); and if available, provide registration information, including the registration number.	N/A
Eligibility criteria	6	Specify characteristics of the sources of evidence used as eligibility criteria (e.g., years considered, language, and publication status), and provide a rationale.	Methods
Information sources	7	Describe all information sources in the search (e.g., databases with dates of coverage and contact with authors to identify additional sources), as well as the date the most recent search was executed.	Methods
Search	8	Present the full electronic search strategy for at least 1 database, including any limits used, such that it could be repeated.	Supplementary Table 2
Selection of sources of evidence	9	State the process for selecting sources of evidence (i.e., screening and eligibility) included in the scoping review.	Methods
Data charting process	10	Describe the methods of charting data from the included sources of evidence (e.g., calibrated forms or forms that have been tested by the team before their use, and whether data charting was done independently or in duplicate) and any processes for obtaining and confirming data from investigators.	Methods and Supplementary Table 3
Data items	11	List and define all variables for which data were sought and any assumptions and simplifications made.	Methods and Supplementary Table 3
Critical appraisal of individual sources of evidence	12	If done, provide a rationale for conducting a critical appraisal of included sources of evidence; describe the methods used and how this information was used in any data synthesis (if appropriate).	N/A
Synthesis of results	13	Describe the methods of handling and summarizing the data that were charted.	Methods
RESULTS			
Selection of sources of evidence	14	Give numbers of sources of evidence screened, assessed for eligibility, and included in the review, with reasons for exclusions at each stage, ideally using a flow diagram.	Results

SECTION	ITEM	PRISMA-ScR CHECKLIST ITEM	Reported in Section
Characteristics of sources of evidence	15	For each source of evidence, present characteristics for which data were charted and provide the citations.	Results and Supplementary Table 4 and 5.
Critical appraisal within sources of evidence	16	If done, present data on critical appraisal of included sources of evidence (see item 12).	N/A
Results of individual sources of evidence	17	For each included source of evidence, present the relevant data that were charted that relate to the review questions and objectives.	Results and Supplementary Table 6 and 7
Synthesis of results	18	Summarize and/or present the charting results as they relate to the review questions and objectives.	Results
DISCUSSION			
Summary of evidence	19	Summarize the main results (including an overview of concepts, themes, and types of evidence available), link to the review questions and objectives, and consider the relevance to key groups.	Discussion
Limitations	20	Discuss the limitations of the scoping review process.	Discussion
Conclusions	21	Provide a general interpretation of the results with respect to the review questions and objectives, as well as potential implications and/or next steps.	Discussion
FUNDING			
Funding	22	Describe sources of funding for the included sources of evidence, as well as sources of funding for the scoping review. Describe the role of the funders of the scoping review.	Acknowledgment

Supplementary Table 2: Search Strategy (list of Queries used to search various databases)

Database	Query
**PubMed**	(("machine learning"[Mesh] OR "deep learning"[Mesh] OR "artificial intelligence"[Mesh] OR "machine learning"[Title/Abstract] OR "deep learning"[Title/Abstract] OR "artificial intelligence"[Title/Abstract] OR "neural network*" [Title/Abstract] OR "Convolutional neural network*" [Title/Abstract] OR "CNN" [Title/Abstract] OR "Artificial neural network*" [Title/Abstract] OR "supervised learning"[Title/Abstract] OR "unsupervised learning"[Title/Abstract] OR "ensemble learning" [Title/Abstract] OR "Support vector machine" [Title/Abstract] OR "Naïve Bayes" [Title/Abstract] OR "decision tree" [Title/Abstract] OR "Random Forest" [Title/Abstract] OR "Naive Bayes" [Title/Abstract] OR "clustering" [Title/Abstract] OR "K- Nearest Neighbor*" [Title/Abstract] OR "K-means" [Title/Abstract] OR "Long Short-Term Memory Networks" [Title/Abstract] OR "Autoencoder*" [Title/Abstract] OR "Generative Adversarial Network" [Title/Abstract] OR "GAN*" [Title/Abstract]) AND ("diabetes"[Title/Abstract] OR "T2D*"[Title/Abstract] OR (type 2[Title/Abstract] AND DM[Title/Abstract])) AND ("prognosis"[Title/Abstract] OR "prediction"[Mesh] OR "predict*"[Title/Abstract] OR "incident" [Title/Abstract]) AND (longitudinal [Mesh] OR longitudinal[Title/Abstract] OR follow-up[Title/Abstract] OR "follow up"[Title/Abstract] OR "retrospective" [Title/Abstract] OR "prospective" [Title/Abstract]) NOT ("Diagnos*"[Title/Abstract]) AND (2000:2022[pdat]) AND (english[Filter]))<br><br>Results: 356 studies
**Scopus**	(TITLE-ABS-KEY ("prognosis") OR TITLE-ABS-KEY ("prediction") OR TITLE-ABS-KEY ("incident")) AND (TITLE-ABS-KEY (longitudinal) OR TITLE-ABS-KEY (follow-up) OR TITLE-ABS-KEY ("follow up") OR TITLE-ABS-KEY ("retrospective") OR TITLE-ABS-KEY ("prospective")) AND (TITLE-ABS-KEY ("diabetes") OR TITLE-ABS-KEY ("T2D*") OR (TITLE-ABS-KEY (type 2) AND TITLE-ABS-KEY (dm))) AND (TITLE-ABS-KEY ("machine learning") OR TITLE-ABS-KEY ("deep learning") OR TITLE-ABS-KEY ("artificial intelligence") OR TITLE-ABS-KEY ("neural network*") OR TITLE-ABS-KEY ("Convolutional neural network*") OR TITLE-ABS-KEY ("CNN") OR TITLE-ABS-KEY ("supervised learning") OR TITLE-ABS-KEY ("unsupervised learning") OR TITLE-ABS-KEY ("ensemble learning") OR TITLE-ABS-KEY ("Support vector machine") OR TITLE-ABS-KEY ("Artificial neural network*") OR TITLE-ABS-KEY ("decision tree") OR TITLE-ABS-KEY ("Random Forest") OR TITLE-ABS-KEY ("Naïve Bayes") OR TITLE-ABS-KEY ("Naive Bayes") OR TITLE-ABS-KEY ("K- Nearest Neighbor*") OR TITLE-ABS-KEY ("clustering") OR TITLE-ABS-KEY ("K-means") OR TITLE-ABS-KEY ("Long Short-Term Memory Networks") OR TITLE-ABS-KEY ("Autoencoder*") OR TITLE-ABS-KEY ("Generative Adversarial Network") OR TITLE-ABS-KEY ("GAN*")) AND (PUBYEAR > 2000) AND NOT (TITLE-ABS-KEY ("diagnos*")) AND (LIMIT-TO (LANGUAGE,"English"))<br><br>Results: 625
**Google Scholar**	(“machine learning” OR “deep learning” OR “artificial intelligence”) AND (“prognosis” OR "prediction") AND (“diabetes” OR “T2D*”) AND ("longitudinal" OR "follow-up" OR "follow up")<br><br>- Results: Top 100 studies
**IEEE Xplore**	("All Metadata":"Artificial intelligence" OR "All Metadata":"Machine learning" OR "All Metadata":"Deep learning" OR "All Metadata":“neural network*” OR "All Metadata":“ensemble learning”) AND ("All Metadata":“follow-up” OR "All Metadata": “follow up” OR "All Metadata":“longitudinal” OR "All Metadata":“retrospective” OR "All Metadata":“prospective”) AND ("Abstract":"diabetes" OR "Abstract":"T2D*") AND ("All Metadata":“predict*” OR "All Metadata": “prognosis” OR "All Metadata":“incident”) NOT ("Abstract":"diagnos*")<br><br>Results: 24 studies

Supplementary Table 3: Data extraction form

Concept	Definition
**Study Characteristics**	
ID	Unique ID assigned to each study
Author	The first author of the study.
Year	The year in which the study was published
Country of publication	The country of the first author' institution.
Publication type	Journal or conference or book chapter
Study aim	The aim of each study
Study design	Type of study design (e.g., randomized control trial, observational study, etc.).
Sample Size	Number of participants used.
Country of origin	The country of the study's participants
AI Model Type	Specific type of AI model used (e.g., neural network, SVM, decision tree, etc.).
Data Source	Source of data for the study (e.g., EHR, medical imaging, multi-omics, etc.).
Modality Approach	Indicates if the study used a unimodal (single source of data) or multimodal (multiple integrated sources of data) approach.
Data Fusion Type	For studies using a multimodal approach, this indicates the type of data fusion used: early (feature-level), late (decision-level), or joint (hybrid).
**Dataset Characteristics**	
Type of data sources	Public or private
Name of the data source if available	The specific name of the dataset or the source
Data source (full URL)	Mention full URL of the dataset if exist
**Evaluation**	
Type of validation	What type of validation the authors used? (e.g., train test split, k-fold cross validation, external validation)
Evaluation metrics	Metrics used to evaluate the model's performance (e.g., AUC, accuracy, precision, etc.).
AUC Value	Represents the Area Under the Receiver Operating Characteristic (ROC) Curve, providing a measure of the model's ability to distinguish between the positive and negative classes.
**Interpretation and risk predictors**	
Interpretability methods	Methods used to interpret the AI model's predictions or decisions.
Risk predictors	The risk factors/biomarkers that the study reported.
**Reproducibility and reporting Standards**	
Code Availability	Whether the study provided open-source code or not.
Reporting Standards Used	The specific reporting guidelines or standards adhered to by the study, such as TRIPOD

Supplementary Table 4: Study's Characteristics

Ref.	Publication type	Country of publication	year	Study aim	Study designs	Follow-up period	How the study ascertained diagnoses of T2DM	Dataset name if exist	Dataset link if public	Reporting standards (Yes/NO)
[1]	Journal	China	2022	Improve T2DM prediction by identifying novel metabolic markers among the Chinese Han ethnicity	case-control	12 years	FPG ≥ 7.0 mmol/L or a history of T2D	N/A	N/A	No
[2]	Journal	China	2021	Establish a T2DM prediction tool and evaluate the potential of Genetic Risk Score (GRS) in a rural Chinese population	prospective cohort study	3 years	self-reported T2DM with current use of oral hypoglycemic medications or insulin, or FPG ≥ 7.0 mmol/L	N/A	N/A	No
[3]	Journal	Estonia	2021	Investigate predictive potential of gut microbiome for T2D development during healthy and pre-diabetic stages	prospective cohort study	1.5, 5 years	N/A	METSIM (Metabolic Syndrome in Men)	https://www.ncbi.nlm.nih.gov/projects/gap/cgi-bin/study.cgi?study_id=phs000743.v1.p1	No
[4]	Journal	USA	2018	Test deep neural network (DNN) to predict T2DM using genetic data	case-control	> 14 years	self-reported	Nurses' Health Study (NHS) and the Health Professionals Follow-up Study (HPFS)	https://dbgap.ncbi.nlm.nih.gov/aa/wga.cgi?page=login	No
[5]	Journal	Finland	2017	Evaluate predictive power of comprehensive metabolomics profiles in predicting future risk of T2DM	retrospective cohort study	10 years	2h-BG after 75 g OGTT≥200 mg/dL (11.1 mmol)	Botnia Prospective Study	https://thl.fi/en/web/thl-biobank/for-researchers/sample-collections/the-botnia-study	No
[6]	Journal	Canada	2016	Identify a metabolomics signature that can predict the transition from gestational diabetes mellitus (GDM) to T2D in women	case-control	2 years	ADA criteria	The Study of Women, Infant Feeding, and Type 2 Diabetes Mellitus After GDM Pregnancy (SWIFT)	https://clinicaltrials.gov/ct2/show/study/NCT01967030	No
[7]	Journal	Japan	2022	Identify patterns of fat distribution associated with different risks of developing T2DM	retrospective case-cohort study	6 years	FPG >=126 mg/dL or HbA1c >= 6.5% (48 mmol/mol) or medications	N/A	N/A	No
[8]	Journal	China	2021	T2DM detection and risk prediction using retina scans and other factors	retrospective	5 years	FPG ≥7.0 mmol/L or HbA1c >=	N/A	N/A	No

							6.5% or a history of drug treatment for diabetes			
[9]	conference	USA	2020	Investigate the role of CT images in the prediction of T2DM onset. Secondly, construct a deep learning framework that combines CT imaging slices with EMR data to refine the prediction of T2DM onset.	retrospective	1 year	ICD-9 codes	N/A	N/A	No
[10]	Journal	USA	2022	Develop and evaluate a non-invasive deep learning algorithm for screening T2DM using retinal images	prospective cohort study	median of 8 years	self-report T2DM or diagnostic codes recorded across all hospital visits.	UK biobank	https://www.ukbiobank.ac.uk/	No
[11]	Journal	China	2022	Predict patients with prediabetes progressing to diabetes based on demographic information and laboratory results	retrospective cohort study	1-year and 2-year	FPG ≥ 7.0 mmol/L or a self-reported T2DM	N/A	N/A	No
[12]	Journal	Canada	2021	Develop and validate a population-level machine learning model for predicting T2DM 5 years before onset using administrative health data	retrospective cohort study	5 years	N/A	N/A		Yes
[13]	Journal	Korea	2021	Investigate the association of CAR with incidence risk of T2DM in adults without chronic disease	retrospective cohort study	7.6 years	FPG ≥126 mg/dL (7.0 mmol/L) or HbA1c ≥6.5% (48 mmol/mol) or 2h-BG after 75 g OGTT≥200 mg/dL (11.1 mmol) or medications	N/A	N/A	No
[14]	Journal	Korea	2021	Develop a deep learning (DL) based model for predicting T2DM in the Korean population	case-control	5, 10 years	ICD-10 codes (E11.x-E14.x) or medications and FBG levels.	National Health Insurance Service-Health Screening (NHIS-HEALS)	https://nhiss.nhis.or.kr/bd/ab/bdaba000eng.do	No
[15]	Journal	Spain	2021	Determine the relationships between inflammatory biomarkers and progression to T2DM	retrospective cohort study	12 years	ADA criteria:<br>FPG levels >126 mg/dL (7.0 mmol/L) or HbA1c >6.5% (>48 mmol/mol)	N/A	N/A	No
[16]	Journal	Australia	2020	Develop a substantially improved diabetes risk prediction model using machine-learning algorithms	retrospective cohort study	3, 5, 7, and 10 years	self-reported T2DM or medications or had gestational diabetes	The 45 and Up Study	https://www.saxinstitute.org.au/solutions/45-and-up-study/	No

[17]	Journal	Singapore	2020	Evaluate the performance of machine learning (ML) algorithms for the prediction of risk of diabetes and other diseases<br>Evaluate the performance of ML algorithms and to compare them with logistic regression for the prediction of risk of diabetes and other diseases	prospective cohort study	6 years	nFPG > 11.1 mmol/l or HbA1c > 6.5% or medications	N/A		No
[18]	Journal	Pakistan	2019	Determine the risk of developing diabetes over different time horizons using irregular and sparsely sampled EMRs data	prospective cohort study	8 years	N/A	Canadian Primary Care Sentinel Surveillance Network (CPCSSN)	http://cpcssn.ca/join-cpcssn/for-researchers/	No
[19]	Journal	Pakistan	2019	Validate the performance of the Framingham Diabetes Risk Scoring Model (FDRSM) using a Hidden Markov Model (HMM)	retrospective cohort study	8 years	N/A	Canadian Primary Care Sentinel Surveillance Network (CPCSSN)	http://cpcssn.ca/join-cpcssn/for-researchers/	No
[20]	Journal	Kuwait	2019	Build prognostic models for the risk of T2DM in the Arab population using machine-learning algorithms and simple non-invasive clinical markers	retrospective cohort study	3, 5 or 7 years	T2DM was ascertained through clinical procedures and was validated by way of using recorded blood glucose levels	N/A	N/A	No
[21]	Journal	South Korea	2019	Develop a T2DM predictive model using electronic medical records (EMRs) and machine learning	retrospective cohort study	5 years	FPGe ≥126 mg/dL or HbA1c ≥6.5%, or medications	N/A	N/A	No
[22]	Journal	Saudi Arabia	2017	Develop a T2DM predictive model using cardiorespiratory fitness data and machine learning	retrospective cohort study	5 years	N/A	N/A	N/A	No
[23]	Journal	Iran	2016	Use decision tree (DT) method for development of different prediction models for incidence of T2D and explore interactions between predictor variables in those models	prospective cohort study	median of 9.5	FPG ≥7 mmol/L or 2h-PCPG ≥11.1 mmol/L or medications	Tehran Lipid and Glucose Study (TLGS)	https://endocrine.ac.ir/page/Tehran-Lipid-and-Glucose-Study-TLGS?lang=en	No
[24]	Journal	USA	2016	Investigate the potential of machine learning methods for accurate prediction of incident diabetes in a high-dimensional setting defined by a large number of predictors	retrospective cohort study	9 years	medications or self-report T2DM or FPG > = 126 mg/dl or HbA1c > = 6.5%	N/A	N/A	No

[25]	Journal	Iran	2016	Predicting diabetes in a prospective cohort of the Tehran Lipid and Glucose Study (TLGS)	prospective cohort study	12 years	FPG >=126 mg/dL or 2h-PCPG >= 200 mg/dL or medications	Tehran Lipid and Glucose Study (TLGS)	https://endocrine.ac.ir/page/Tehran-Lipid-and-Glucose-Study-TLGS?lang=en	NO
[26]	Journal	USA	2015	Develop a population-level risk prediction model for T2DM which can be directly applied to health insurance claims and other readily available clinical and utilization data	retrospective cohort study	1 and 2 years	ICD-9 code, or medication or HbA1c > = 6.5%	N/A	N/A	No
[27]	Journal	Iran	2014	Create a simple tool to predict individuals at low risk for T2DM	prospective cohort study	12 years	FPg >=126 mg/dl or 2h-PCPG >= 200 mg/dl or medications	Tehran Lipid and Glucose Study (TLGS)	https://endocrine.ac.ir/page/Tehran-Lipid-and-Glucose-Study-TLGS?lang=en	No
[28]	Journal	New Zealand	2019	Improve the prediction of the onset of T2DM using EHRs and deep learning	retrospective cohort study	4 years	N/a	Practice fusion dataset	N/A	No
[29]	conference	Netherland	2019	Identify healthy subjects who are at an increased risk of developing T2DM in the future	case-control	average of 7.5 years	FPG ≥126 mg/dL or 2h-PCPG ≥200 mg/dL	San Antonio Heart Study (SAHS).	https://www.ncbi.nlm.nih.gov/projects/gap/cgi-bin/study.cgi?study_id=phs001215.v3.p2	No
[30]	Journal	Italy	2020	Early identification of individuals at risk of developing T2DM using the TyG index	retrospective cohort study	9 years	N/A	FIMMG	https://vrai.dii.univpm.it/content/fimmg-dataset	No
[31]	Journal	Qatar	2020	Identify individuals who are at risk of developing T2DM in the future and provide them with a fair warning	case-control	average of 7.5 years	N/A	San Antonio Heart Study (SAHS).	https://www.ncbi.nlm.nih.gov/projects/gap/cgi-bin/study.cgi?study_id=phs001215.v3.p2	No
[32]	Journal	Qatar	2019	Develop a predictive model using machine learning techniques to identify individuals who are at an increased risk of developing T2DM	case-control	average of 7.5 years	N/A	San Antonio Heart Study (SAHS).	https://www.ncbi.nlm.nih.gov/projects/gap/cgi-bin/study.cgi?study_id=phs001215.v3.p2	No
[33]	Journal	USA	2012	Test the feasibility of using data collected in electronic medical records for development of effective models for diabetes risk forecasting	case-control	6 months, 1- year	ICD-9 codes or HbA1c > = 6.5, or at least two random blood glucose readings over 200 mg/dL	Synthetic Derivative (SD)	https://www.vumc.org/main/home	No

[34]	Journal	Greece	2021	Develop and validate a diabetes risk assessment score for healthy/undiagnosed participants	case-control	2 years	N/A	English Longitudinal Study of Ageing (ELSA)	https://www.elsa-project.ac.uk/accessing-elsa-data	No
[35]	Journal	Pakistan	2020	Predict the risk of developing T2DM in a particular individual based on their clinical measurements	retrospective cohort study	8 years	FBG >=126 mg/dL or HbA1c>=6.5	Canadian Primary Care Sentinel Surveillance Network (CPCSSN)	http://cpcssn.ca/	No
[36]	Journal	Spain	2022	Develop a simple and practical decision model to predict the risk of incident T2DM among the general population	prospective cohort study	7.5 years+-0.6 years	random blood sugar level ≥200 mg/dl in the presence of diabetes symptoms (polyuria, polydipsia or unexplained weight loss); FPG ≥126 mg/dl or HbA1c ≥6.5% or 2h-BG after 75 g OGTT	The Di@bet.es Study	https://www.sediabetes.org/cientifico-y-asistencial/investigacion/proyectos-de-investigacion/estudio-dibet-es/	Yes
[37]	conference	Japan	2015	study disease prediction using periodical health checkup data, daily monitoring to maintain a healthy condition, and early life disease detection	case-control	1 year	HbA1c ≥6.5%	N/A	N/A	No
[38]	Journal	Korea	2021	Predict the occurrence of T2DM in the following year using variables in the current year	case-control	1-4 years	FPG >125 mg/dl	N/A	N/A	No
[39]	Journal	China	2021	Establish and validate a risk assessment system that combines demographic and clinical variables to predict the 3-year risk of incident diabetes in Chinese adults	retrospective cohort study	3 years	FPG ≥7.00 mmol/L or self-reported T2DM	DATADRYAD.	https://datadryad.org/stash/dataset/doi:10.5061%2Fdryad.ft8750v	Yes
[40]	Journal	Korea	2022	Develop a machine learning-based prediction model for identifying individuals at high risk of developing T2DM using genome-wide polygenic risk score and metabolic profiles	prospective cohort study	8.3 ± 2.8 years	self-reported T2DM or medications or meeting the ADA diagnostic criteria (FPG ≥7.0 mmol/L or 2-h BG ≥11.1 mmol/L or HbA1c ≥48 mmol/mol (6.5%))	N/A		No

Supplementary Table 5: Study's demographic characteristics, AI models, and performance metrics. ***Abbreviations:*** RF: Random Forest; ANN: Artificial Neural Network; GBM: Gradient Boosting Machine; DNN: Deep Neural Network; LR: Logistic Regression; RLS: Regularised Least Squares; J48 DT: J48 Decision Tree; NB: Naive Bayes; CNN: Convolutional Neural Network; MLP: Multilayer Perceptron; ResNet18: Residual Neural Network; LSTM: Long Short-Term Memory; Cox regression: Cox Proportional Hazards Model; LDA: Linear Discriminant Analysis; CART: Classification and Regression Tree; SVM: Support Vector Machine; KNN: K-Nearest Neighbors; FFNN: Feed-Forward Neural Network; HMM: Hidden Markov Model; QDA: Quadratic Discriminant Analysis; LMT: Logistic Model Tree; RG: Ridge Regression; QUEST: Quick Unbiased Efficient Statistical Tree; C5.0: C5.0 Decision Tree (Commercial version of the C4.5 algorithm); PNN: Probabilistic Neural Network; DL: Deep Learning; MIL-Boost: Multiple Instance Learning - Boost; A1DE: Averaged One-Dependence Estimators; A2DE: Averaged Two-Dependence Estimators; AdaBoost: Adaptive Boosting; SVM-RBF: Support Vector Machine with Radial Basis Function kernel; WeightedVotingLRRFs: Weighted Voting of LR and RF models; CHAID: Chi-Square Automatic Interaction Detector; XGBoost: Extreme Gradient Boosting; AUC: area under the receiver operating curve; NRI: net reclassification improvement; IDI: integrated discrimination improvement; G-mean: geometric mean; cNRI: category-free NRI.

Ref.	Country of origin	Ethnicity	Sample size	Male %	AI models	Discrimination metric	Calibration metrics
[1]	China	Han ethnicity	440	41.8	RF	AUC, Sensitivity, Specificity	N/A
[2]	China	N/A	5,712	36	ANN, RF, GBM	AUC, NRI	Brier Score
[3]	Finland	Finish	608	100	RF	N/A	N/A
[4]	USA	European	5,828	43	DNN, LR	AUC, NRI, IDI	N/A
[5]	Finland	Finish	543	50.46	RLS	AUC, IDI	N/A
[6]	USA/California	Non-Hispanic white, Asian (East, South, Southeast), Non-Hispanic black, Hispanic	244	0	J48 DT, LR, NB	AUC, Accuracy, Sensitivity, Specificity, Precision, F1	N/A
[7]	Japan	N/A	754	87.7	K-means	N/A	N/A
[8]	China	multi-ethnicity	8157	50.90	RF & LR for Clinical data, CNN for Fundus images, MLP for Fusion	AUC, Sensitivity, Specificity	N/A
[9]	USA	N/A	997	N/A	CNN+ MLP	Accuracy, AUC, F1, Sensitivity, Precision	N/A
[10]	United Kingdom	White, Asian, Black, Mixed, others	62,262	N/A	ResNet18	AUC, NRI	N/A
[11]	China	N/A	12,009	45	LR, RF, DT, XGBoost	AUC, Accuracy, Sensitivity, Specificity, F1	N/A
[12]	Canada/Ontario	N/A	1,893,901	48	XGBoost	AUC	N/A
[13]	South Korea	N/A	5,904	46	RF	AUC	N/A
[14]	South Korea	N/A	335,302	55.82	LSTM, Cox regression	AUC, Sensitivity, Specificity,	Hosmer-Lemeshow test
[15]	Spain	N/A	1,576	49.1	LDA, CART, SVM, KNN	Accuracy, AUC, Sensitivity, Specificity,	N/A
[16]	Australia	N/A	236,684	45	LR, RF, FFNN, GBM	AUC	N/A
[17]	Singapore	Asian Chinese, Indian, and Malay	4,992	47.4	FFNN, RF, SVM, GBM, KNN, LR	AUC	N/A
[18]	Canada	N/A	1981	39	HMM	AUC	N/A
[19]	Canada	N/A	911	38.9	HMM	AUC	N/A
[20]	Kuwait	Native Arab from Kuwait	1,837	49.5	LR, KNN, SVM	AUC	N/A
[21]	South Korea	N/A	8454	46.9	LR, LDA, QDA, KNN	AUC	N/A
[22]	USA	N/A	32,555	55.6	LR, NB, J46 DT, RF, LMT, Ensemble (NB, RG, LMT)	AUC, Accuracy, Sensitivity, Specificity, Precision, F1	N/A

[23]	Iran	Almost Iranian	6647	43.40	DT (CART, QUEST, C5.0)	AUC, Sensitivity, Specificity, G-Mean, F1	N/A
[24]	USA	African Americans	3633	36.50	LR, RF	AUC, Accuracy, Sensitivity, Specificity	N/A
[25]	Iran	Almost Iranian	6647	43.4	DT, NB, PNN	Accuracy, Sensitivity, Specificity, precision, F1	N/A
[26]	USA	N/A	793153	45	Sparse, or L1-regularized LR	AUC	N/A
[27]	Iran	Almost Iranian	6647	43.30	DT	Accuracy, Sensitivity, Specificity, F1, Precision	N/A
[28]	USA	N/A	9948	43	DL	AUC, Accuracy, Sensitivity, Specificity	N/A
[29]	USA	Mexican-American and non-Hispanic white ethnicity	1496	N/A	SVM	Accuracy, Sensitivity, Specificity	N/A
[30]	Italy	N/A	256	49	MIL-Boost	AUC, Accuracy, Sensitivity, Precision, F1	N/A
[31]	USA	Mexican-American and non-Hispanic white ethnicity.	1368	N/A	SVM, Ensemble (A1DE, A2DE, RF, AdaBoost, Bagging, Boosting)	AUC, Accuracy, Sensitivity, Specificity	N/A
[32]	USA	Mexican-American and non-Hispanic white ethnicity	1492	N/A	linear SVM, SVM-RBF	Accuracy, Sensitivity, Specificity, g-mean	N/A
[33]	USA	N/A	2,280	40	LR, NB, CART, RF, SVM	AUC, Sensitivity, Specificity	N/A
[34]	English population	N/A	2009	46.6	NB, DT, RF, LR, Ensemble (WeightedVotingLRRFs)	AUC, Sensitivity, Specificity	N/A
[35]	Canada	N/A	1918	38.9	HMM	AUC	N/A
[36]	Spain	N/A	2408	39.7	DT (CHAID)	Accuracy	N/A
[37]	Japan	N/A	9605	N/A	DT	AUC	N/A
[38]	South Korea	N/A	253,395	N/A	LR, RF, SVM, XGBoost, Ensemble Learning	Accuracy, Sensitivity, Precision, F1	N/A
[39]	China	N/A	15928	64.70	XGBoost	AUC	calibration plot
[40]	South Korea	N/A	1425	45.20	RF, LR	AUC, NRI, cNRI, IDI	Brier score, calibration plot

Supplementary Table 6: Unimodal studies. ***Abbreviations*:** WC: Waist circumference, total cholesterol (TC), triglyceride (TG), high density lipoprotein cholesterol (HDL-C), LDL-C: low density lipoprotein cholesterol, Alanine aminotransferase (ALT), aspartate aminotransferase (AST), CAR: C-reactive protein-to-albumin ratio, GGT: gamma-glutamyl transferase, systolic blood pressure (SBP), diastolic blood pressure (DBP), and body mass index (BMI), High-sensitivity C-reactive protein (hs-CRP), 2-hour post challenge plasma glucose (2h-PCPG), Mean arterial blood pressure(MAP),Area under glucose curve (AuG),body's glucose absorption index (BGAI), body's insulin production index (BIPI), serum glutamic-oxaloacetic transaminase (GOT), Gamma-glutamyl transferase (GGT), gamma-glutamyl transpeptidase (GGTP),serum urea nitrogen (BUN), serum creatinine (Scr), Body Surface Area (BSA).

Ref.	Data modality	AI models	Best AI model	Performance	Validation Type	Reported risk factors
[11]	EHR: sociodemographic (education level); lifestyle (smoking, alcohol drinking, physical activity); anthropometric measurements (BMI, WC); Glycemic traits (FPG); blood lipids (TC, HDL-C, LDL-C, TG); liver enzymes (ALT);	LR, RF, DT, and XGBoost	XGBoost	AUC= 0.6742 (1-year)<br>AUC = 0.6707(2-years)	Internal (Hold-out)	FPG, TG, WC, TC, Education, BMI, ALT
[12]	EHR: demographic information, laboratory measurements, drug benefits, health care system interactions, social determinants of health, and ambulatory care and hospitalization records	XGBoost	XGBoost	AUC=0.80	Internal (Train-valid-test)	N/A
[13]	EHR: Sociodemographic (age, sex); FHD; insulin resistance (HOMA-IR); anthropometric measurements (BMI); Glycemic traits (glucose level); blood lipids (TC, HDL-C, TG); Inflammatory biomarkers (CAR);	RF	RF	AUC=0.555	Internal (Hold-out)	N/A
[14]	EHR: Sociodemographic (age, sex); lifestyle (smoking, alcohol drinking, physical activity); anthropometric measurements (BMI); glycemic traits (FPG, HbA1C); blood lipids (TC); liver enzymes (ALT, AST); other biomarkers (GGT, Proteinuria); FHD; medical history of disease (hypertension, Heart disease, Strok, others), blood pressure (SBP, DBP).	LSTM, and Cox regression	LSTM	AUC = 0.827 (5-years)<br>AUC = 0.807 (10-years)	Internal (Hold-out)	FPG, age, sex, ALT, BMI, GGT, SBP, TG, AST, Alcohol intake
[15]	EHR: Sociodemographic (age, sex, education, income, residential rurality, insurance, marital status); insulin resistance (HOMA-IR); anthropometric measurements (BMI); Inflammatory biomarkers (log(hs-CRP), and Fibrinogen)	LDA, CART, SVM, and KNN	CART	AUC= 0.90	Internal (K-fold cross-validation)	HOMA-IR, fibrinogen, hs-CRP

[16]	EHR: Sociodemographic (age, sex); anthropometric measurements (BMI); FHD, lifestyle (smoking, alcohol drinking, physical activities, sleeping, dietary indicators,) blood lipids (Dyslipidemia); psychological factors (depression); medical history of disease (cancer, CVD, hypertension)	LR, RF, FFNN, and GBM	RF and GBM	AUC for 3-years prediction:<br>RF= 0.7868<br>GBM= 0.7927<br><br>AUC for 5-years prediction:<br>RF= 0.7769<br>GBM= 0.7769<br><br>AUC for 7-years prediction:<br>RF= 0.7531<br>GBM= 0.7589<br><br>AUC for 10-years prediction:<br>RF= 0.7439<br>GBM= 0.7491	Internal (Train-valid-test)	BMI, age, hypertension, health insurance, income, FHD, Education, alcohol intake, CVD, dyslipidemia
[17]	EHR: Sociodemographic (age, sex, ethnicity, income education); anthropometric measurements (BMI); lifestyle (smoking, alcohol drinking,) blood lipids (LDL-C, HDL-C); Glycemic traits (non FPG,); FHD; Medications (antihypertensive drug, anti-cholesterol drug); other biomarkers (adiponectin, aldosterone, Leptin, sign of retinopathy, ocular vascular calibers (arteriolar and venular))	FFNN, RF, SVM, GBM, KNN, and LR	LR	AUC = 0.768	internal (Hold-out)	BGL, BMI, FHD
[18]	EHR: Sociodemographic (age, sex); anthropometric measurements (BMI); blood pressure (SBP); Glycemic traits (FPG, HbA1c); blood lipids (HDL-C, LDL-C, TG); psychological factors (depression frequency)	HMM	HMM	AUC = ~0.74	internal (Hold-out)	TC, FPG, TG, HbA1c, HDL, LDL, BMI, SBP, age
[19]	EHR: Sociodemographic (age); anthropometric measurements (BMI); Blood pressure (SBP); Glycemic traits (FPG); Blood lipids (HDL-C, TG)	HMM	HMM	AUC = 0.869	Internal (leave one out cross validation)	BP, FPG, TG, HDL, BMI, age
[20]	EHR: Sociodemographic (age, sex); Anthropometric measurements (BMI); FHD; medical history of disease (hypertension)	LR, KNN, and SVM	KNN	AUC = 0.83 (3-year)<br>AUC = 0.82 (5-year)<br>AUC = 0.79 (7-year)	internal (K-fold cross-validation)	Age, BMI, FHD, Family history of hypertension, pre-existing hypertension, sex

[21]	EHR: HbA1C, age, glucose, Diuretics, Statins, Angiotensin receptor blockers (ARBs), Hypertension, Clopidorel, calcium channel blockers (CCB): dihydropyridine (DHP), Nitrates, Non-selective beta blockers (BB), coronary artery disease (CAD), CCB: non-DHP, Stroke, Selective BB, Hyperuricemia, percutaneous coronary intervention (PCI), chronic kidney disease–the modification of diet in renal disease (CKD-MDRD) state, Cilostazol, Proton pump inhibitors	LR, LDA, QDA, KNN	LR	AUC =0.78	Internal (K-fold cross-validation)	N/A
[22]	EHR: demographics (age, ethnicity); lifestyle (sedentary life); medical history of diseases (obesity, history of premature coronary artery, hypertension); Blood lipids (Hyperlipidemia); Blood pressure (SBP, DBP);<br>Medications (Aspirin); Other biomarkers (Heart Rate (HR))	LR, NB, J46 DT, RF, Logistic Model Tree (LMT), and ensemble voting	Ensemble voting	AUC= 0.922	Internal (K-fold cross-validation)	N/A
[23]	EHR: (1) Age, FBG, WC, BMI, Waist-to-hip ratio, Waist-to-height ratio, Cholesterol-to-HDL ratio, Triglyceride-to-HDL ratio, Pulse pressure, MAP, Glomerular filtration rate, Total length of stay in the city (years), Use of the ACE inhibitors, Current status of pregnancy, aspirin, educational level, FHD, family history of premature cardiovascular diseases.<br><br>(2) 2h-PCPG + (1)	DT algorithms (CART, QUEST, and C5.0)	QUEST	AUC for males = 0.80<br>AUC for females = 0.81	Internal (Hold-out)	FPG, 2h-PCPG, WHtR, MAP
[24]	EHR: Sociodemographic (age); Anthropometric measurements (BMI, WC, BSA); Glycemic traits (FPG, HbA1); blood lipids (TG, HDL-C, LDL-C); other biomarkers (hs-CRP, adiponectin, aldosterone, Leptin, BSA, eGFR<br>); echocardiogram data (2D calculated left ventricular mass)	LR and RF	RF	AUC: 0.82	Internal (Hold-out)	HbA1c, FPG, WC, adiponectin, BMI, hs-CRP, TG, leptin, left ventricular mass, HDL, aldosterone.
[25]	EHR: Sociodemographic (age, sex, education, marital status); lifestyle (smoking, physical activity); anthropometric measurements (BMI, WC, height); Glycemic traits (FPG, 2h-PCPG);	DT, NB, and PNN	NB	AUC = N/A<br>Sensitivity = 0.776<br>Specificity = 0.784<br>Accuracy = 0.783	Internal (Hold-out)	N/A

	Blood lipids (TG, HDL-C); other biomarkers (Creatinine); FHD; medical history of disease (hypertension, history of ischemic heart disease); Blood pressure (SBP); Medications (antihypertensive drugs, lipid-lowering drug)			F- score = 0.440<br>Precision = 0.307<br>Youden's index = 0.56		
[26]	EHR: Not reported in detail	LR	LR	AUC = 0.8	Internal (Hold-out)	HbA1c, TC, TG, ALT, Carbondioxide, disease history (Abnormal glucose, Impaired fasting glucose, Hypertension, Chronic liver disease, Obesity Obstructive sleep apnea, Hypersomnia with sleep apnea, Abnormal blood chemistry, Hyperlipidemia, Anemia, Hypothyroidism, Acute bronchitis), Medications (Metformin, antiarthritics, nonsteroidal-inflammatory drugs)
[27]	EHR: Sociodemographic (age, sex, education, marital status, occupation); lifestyle (physical activity); anthropometric measurements (height, weight, WTH, WC, Hip circumference, WHtR, WHR, BMI); glycemic traits (FPG); Blood lipids(TG,HDL-C,TC, cholesterol to HDL ratio, TG to HDL); Other biomarkers(Creatinine);	DT	DT	AUC = N/A<br>Sensitivity = 0.311<br>Specificity = 0.979<br>Accuracy = 0.905<br>F1 score = 0.95<br>Precision= 0.92	Internal (Hold-out)	FPG, BMI, TG, MAP, Occupation, Education, FHD
[28]	Not reported in detail	DL	DL	AUC = 0.84,	Internal (K-fold cross-validation)	N/A
[29]	EHR: Sociodemographic (age, ethnicity); Glycemic traits (AuG0−120, PG120);	SVM	SVM	Accuracy = 0.89	Internal (K-fold cross-validation)	N/A
[30]	Not reported in detail	MIL-Boost	MIL-Boost	AUC = 0.89	Internal (K-fold cross-validation)	TG, ALT, GGT, Hematocrit, HDL, AST, Urea, DBP, Sodium, Albumin
[31]	Not reported in detail	SVM, ensemble of NB variants (averaged one-	ensemble of NB	AUC= 0.963	Internal (K-fold cross-validation)	N/A

		dependence estimators (A1DE) and averaged two-dependence estimators (A2DE)) , RF, AdaBoost, bagging, and boosting,				
[32]	EHR: Demographics, personal information, and plasma glucose and insulin concentrations recorded before glucose intake and at three time-points thereafter (30, 60, and 120 min)	linear SVM and SVM-RBF	SVM-RBF	AUC = N/A	Internal (K-fold cross-validation)	AuG0-120, ΔG120-0,ΔG120-60,ΔG30-0
[33]	Not reported in detail	LR, NB,CART, RF, and SVM	RF	1- year prediction: AUC = 0.803 (1-year) AUC = 0.804 (half year)	Internal (K-fold cross-validation)	N/A
[34]	Demographics, vital signs and lab tests (Not reported in detail)	NB, DT, RF, LR, ensemble model (Weighted Voting of LR and RFs)	Ensemble model	AUC = 0.884	Internal (Hold-out)	N/A
[35]	EHR: Sociodemographic (age, sex); anthropometric measurements (BMI); Glycemic traits (hba1c); Blood lipids (TC, TG, LDL-C, GDL-C)	HMM	HMM	AUC = 0.804	Internal (Hold-out)	HbA1c, FPG, BMI, TG, age
[36]	EHR: Sociodemographic (age, sex); insulin resistance (HOMA-IR); Glycemic traits (FPG); anthropometric measurements (BMI, WC); blood lipids (TG)	DT using the CHAID method	DT using the CHAID method	AUC = N/A Accuracy = 0.935	Internal (cross-validation)	FPG, TG, age, sex, WC, BMI
[37]	EHR: Anthropometric measurements (BMI); Blood lipids (TG, HDL-C, LDL-C); Blood pressure (SBP); other biomarkers (GTP, creatinine, GOT, GPT, Urine acid)	DT	DT	AUC = 0.63	Internal (Hold-out)	N/A
[38]	EHR: Sociodemographic (age, sex); Glycemic traits (FPG, HbA1C); Anthropometric measurements (BMI); Blood lipids (TG); lifestyle (smoking, physical activity, alcohol drinking); FHD; other biomarkers (Urine acid, GGT);	LR, RF, SVM, XGBoost, ensemble learning	Ensemble learning	AUC = N/A	Internal (K-fold cross-validation)	FPG, BMI, GGTP, TG, sex, age, uric acid, HbA1c, smoking, alcohol intake, physical activity, FHD

[39]	EHR: age, gender, smoking and drinking status, family history of diabetes, body mass index (BMI), systolic blood pressure (SBP), diastolic blood pressure (DBP), fasting plasma glucose (FPG), total cholesterol (TC), triglyceride (TG), low density lipoprotein cholesterol (LDL-C), high density lipoprotein cholesterol (HDL-C), serum urea nitrogen (BUN), serum creatinine (Scr), alanine aminotransferase (ALT)	XGBoost	XGBoost	AUC = 0.910	External and internal (Hold-out)	FPG, BMI, age, HDL, ALT, LDL
[7]	Imaging: CT				Internal (Hold-out)	N/A

Supplementary Table 7: Multimodal studies

Ref.	Data modalities	Fusion strategy	AI models	Best AI model	Performance	Single modality comparison	Validation Type	Reported risk factors
[1]	EHR/ metabolomics and genetics	early	RF	RF	AUC:<br>EHR = 0.798,<br>Genetics = 0.586<br>Genetics + Metabolites = 0.958<br>Metabolites + Genetics+ EHR = 0.960	Yes.	Internal :10-fold cross-validation	5 metabolites (riboflavin, cnidioside A, 2-methoxy-5-(1H-1, 2, 4-triazol5-yl)- 4-(trifuoromethyl) pyridine, 7-methylxanthine, and mestranol)
[2]	EHR/metabolomics and genetics	early	ANN, RF, GBM	GBM	AUC:<br>Clinical = 0.851<br>Genetics+ clinical factors =0.885	Yes.	Internal: Hold-out	N/A
[3]	EHR/microbiome	early	RF	RF	-	Yes. Unclear	Internal: Hold-out	N/A
[4]	EHR/genomics	early	DNN, LR	DNN	AUC:<br>96-SNPs + clinical = 0.831 (in male)<br><br>96-SNPs + clinical = 0.865 (in female)<br><br>678-SNPs = 0.931 (in male)<br><br>678-SNPs = 0.928 (in female)<br><br>678-SNPs + clinical = 0.948 (in male)<br><br>678-SNPs + clinical = 0.946 (in female)	Yes	Internal: Hold-out	N/A
[5]	EHR/metabolomics	early	RLS - Regularized	RLS - Regularized least squares	AUC:<br>clinical risk factors = 0.68	Yes	External and Internal (10-fold cross-validation)	Metabolites (glucose, mannose, α-HB, α-tocopherol,[Hyp3]-BK, X-12063,X-13435)

			least squares		clinical + metabolome = 0.76<br>selected metabolome features = 0.75<br>selected metabolome features + clinical features = 0.78			
[6]	EHR/ metabolomics	early	J48 DT, LR, NB	J48 DT	AUC:<br>metabolites = 0.769<br>glucose model = 0.732<br>fusion model = 0.754	Yes	Internal: cross-validation	Prediction revealed two novel metabolites, PC ae C40:5and SM (OH) C14:1
[40]	EHR/ metabolomics and genomics	early	RF,LR	RF	AUC:<br>EHR = 0.84<br>EHR + genetics = 0.876<br>EHR + genetics +metabolomics = 0.8835	Yes	Internal: 10-fold cross-validation	N/A
[8]	EHR/fundus images	Joint	Clinical data: RF & LR, fundus: CNN, fusion: MLP	Clinical data: RF & LR, fundus: CNN, fusion: MLP	AUC:<br>Fundus-only = 0.820<br>Clinical-only = 0.762<br>Fusion = 0.845	Yes	External and Internal (Hold-out)	Retinal biomarkers such as such as vascular tortuosity, venous dilatation, retinal haemorrhage and cotton wool spots
[9]	EHR/CT images	Joint	CNN+ MLP	CNN+ MLP	AUC:<br>EHR-only = 82.17<br>Images-only = 84.85<br>Fusion = 89.10	Yes	External and Internal (5-fold cross-validation)	N/A
[10]	EHR/fundus images	Joint	ResNet18	ResNet18	AUC:<br>Clinical-only = 0.81<br>Fundus-only = 0.73<br>Fusion-only = 0.844	Yes	External and Internal (Train-valid-test)	N/A

Supplementary Table 8: External Validation Study's Details

Article ID	The type of population studies used for external validation	Country of Participants	Differences vs development data	Study Name	Sample size	Male %	Ethnicity
[5]	1044 participants in the DESIR study from central western France, of whom 231 progressed to T2DM during a 9-year follow-up period	France	Different country	The DESIR study (Data from an Epidemiological Study on the Insulin Resistance Syndrome)	1044	N/A	N/A
[7]	The study used a cohort of 319 individuals without diabetes who underwent MRI and metabolic phenotyping in Germany	Germany	Different country	TDFS: Tubingen Diabetes Family Study	319	27.30%	N/A
[8]	The first external validation dataset consisted of 8,059 individuals from Guangdong Province, China. The second external validation dataset was from Beijing, China and contained 3,376 individuals	China	Same country, several different geographic areas (external test sets 1 and 2)	N/A	Dataset 1: 8,059<br>Dataset 2: 3,376	Dataset1: 55.1%<br>Dataset2: 47.9%	N/A
[10]	6575 retinal fundus images from the University-affiliated Diabetes Center of St. Vincent's Hospital	Australia	Different country	The University-affiliated Diabetes Center of St. Vincent's Hospital	6575	N/A	N/A
[10]	A cohort of 11,113 Japanese participant	Japanese	Different country	The NAGALA (NAfd in the Gifu Area, Longitudinal Analysis) database	11,113	N/A	N/A

References

1. Liu, J., et al., *Metabolic and genetic markers improve prediction of incident type 2 diabetes: A nested case-control study in Chinese.* The Journal of Clinical Endocrinology & Metabolism **107**(11), 3120-3127 (2022)
2. Wang, Y., et al., *Genetic risk score increased discriminant efficiency of predictive models for type 2 diabetes mellitus using machine learning: cohort study.* Frontiers in public health **9,** 606711. (2021)
3. Aasmets, O., et al., *Machine learning reveals time-varying microbial predictors with complex effects on glucose regulation.* Msystems **6**(1), e01191-20 (2021)
4. Kim, J., et al., *Genetic prediction of type 2 diabetes using deep neural network.* Clinical genetics **93**(4), 822-829 (2018)
5. Peddinti, G., et al., *Early metabolic markers identify potential targets for the prevention of type 2 diabetes.* Diabetologia **60**(9), 1740-1750 (2017)
6. Allalou, A., et al., *A predictive metabolic signature for the transition from gestational diabetes mellitus to type 2 diabetes.* Diabetes **65**(9), 2529-2539 (2016)
7. Yamazaki, H., et al., *Fat distribution patterns and future type 2 diabetes.* Diabetes **71**(9), 1937-1945 (2022)
8. Zhang, K., et al., *Deep-learning models for the detection and incidence prediction of chronic kidney disease and type 2 diabetes from retinal fundus images.* Nature Biomedical Engineering **5**(6), 533-545 (2021)
9. Tang, Y., et al. *Prediction of type II diabetes onset with computed tomography and electronic medical records*. in *Multimodal Learning for Clinical Decision Support and Clinical Image-Based Procedures: 10th International Workshop, ML-CDS 2020, and 9th International Workshop, CLIP 2020, Held in Conjunction with MICCAI 2020, Lima, Peru, October pp. 4–8, (2020),* Proceedings 9, pp. 13–23 (2020). Springer
10. Yun, J.-S., et al., *A deep learning model for screening type 2 diabetes from retinal photographs.* Nutrition, Metabolism and Cardiovascular Diseases **32**(5), 1218-1226 (2022)
11. Liu, Q., et al., *Predicting the 2-Year Risk of Progression from Prediabetes to Diabetes Using Machine Learning among Chinese Elderly Adults.* Journal of Personalized Medicine **12**(7), 1055 (2022)
12. Ravaut, M., et al., *Development and validation of a machine learning model using administrative health data to predict onset of type 2 diabetes.* JAMA network open **4**(5), e2111315-e2111315 (2021)
13. Cho, A.-R., et al., *C-reactive protein-to-albumin ratio and 8-year incidence of type 2 diabetes: the Korean genome and epidemiology study.* Acta Diabetologica **58**(11), 1525-1532 (2021)
14. Rhee, S.Y., et al., *Development and validation of a deep learning based diabetes prediction system using a nationwide population-based cohort.* Diabetes & Metabolism Journal **45**(4), 515-525 (2021)
15. Garcia-Carretero, R., L. Vigil-Medina, and O. Barquero-Perez, *The use of machine learning techniques to determine the predictive value of inflammatory biomarkers in the development of type 2 diabetes mellitus.* Metabolic Syndrome and Related Disorders **19**(4), 240-248 (2021)
16. Zhang, L., et al., *Predicting the development of type 2 diabetes in a large Australian cohort using machine-learning techniques: longitudinal survey study.* JMIR medical informatics **8**(7), e16850 (2020)
17. Nusinovici, S., et al., *Logistic regression was as good as machine learning for predicting major chronic diseases.* Journal of clinical epidemiology **122,** 56-69 (2020)
18. Perveen, S., et al., *A hybrid approach for modeling type 2 diabetes mellitus progression.* Frontiers in Genetics **10,** 1076 (2020)
19. Perveen, S., et al., *Prognostic modeling and prevention of diabetes using machine learning technique.* Scientific reports **9**(1), 13805 (2019)
20. Farran, B., et al., *Use of non-invasive parameters and machine-learning algorithms for predicting future risk of type 2 diabetes: a retrospective cohort study of health data from Kuwait.* Frontiers in endocrinology **10,** 624 (2019)
21. Choi, B.G., et al., *Machine learning for the prediction of new-onset diabetes mellitus during 5-year follow-up in non-diabetic patients with cardiovascular risks.* Yonsei medical journal **60**(2), 191-199 (2019)

22. Alghamdi, M., et al., *Predicting diabetes mellitus using SMOTE and ensemble machine learning approach: The Henry Ford ExercIse Testing (FIT) project.* PloS one **12**(7), e0179805 (2017)
23. Ramezankhani, A., et al., *Decision tree-based modelling for identification of potential interactions between type 2 diabetes risk factors: a decade follow-up in a Middle East prospective cohort study.* BMJ open **6**(12), e013336 (2016)
24. Casanova, R., et al., *Prediction of incident diabetes in the Jackson Heart Study using high-dimensional machine learning.* PloS one **11**(10), e0163942 (2016)
25. Ramezankhani, A., et al., *The impact of oversampling with SMOTE on the performance of 3 classifiers in prediction of type 2 diabetes.* Medical decision making **36**(1), 137-144 (2016)
26. Razavian, N., et al., *Population-level prediction of type 2 diabetes from claims data and analysis of risk factors.* Big Data **3**(4), 277-287 (2015)
27. Ramezankhani, A., et al., *Applying decision tree for identification of a low risk population for type 2 diabetes. Tehran Lipid and Glucose Study.* Diabetes research and clinical practice **105**(3), 391-398 (2014)
28. Nguyen, B.P., et al., *Predicting the onset of type 2 diabetes using wide and deep learning with electronic health records.* Computer methods and programs in biomedicine **182,** 105055 (2019)
29. Abbas, H., et al. *Predicting diabetes in healthy population through machine learning*. in *2019 IEEE 32nd International Symposium on Computer-Based Medical Systems (CBMS)* IEEE (2019)
30. Bernardini, M., et al., *Early temporal prediction of type 2 diabetes risk condition from a general practitioner electronic health record: a multiple instance boosting approach.* Artificial Intelligence in Medicine **105,** 101847 (2020)
31. Islam, M.S., et al., *Advanced techniques for predicting the future progression of type 2 diabetes.* IEEE Access **8,** 120537-120547 (2020)
32. Abbas, H.T., et al., *Predicting long-term type 2 diabetes with support vector machine using oral glucose tolerance test.* Plos one **14**(12), e0219636 (2019)
33. Mani, S., et al. *Type 2 diabetes risk forecasting from EMR data using machine learning*. in *AMIA annual symposium proceedings* , vol. 2012, p. 606 (2012)
34. Fazakis, N., et al., *Machine learning tools for long-term type 2 diabetes risk prediction.* IEEE Access **9,** 103737-103757 (2021)
35. Perveen, S., et al., *Handling irregularly sampled longitudinal data and prognostic modeling of diabetes using machine learning technique.* IEEE Access **8,** 21875-21885 (2020)
36. Martínez-Hervás, S., et al., *Developing a simple and practical decision model to predict the risk of incident type 2 diabetes among the general population: The Di@ bet. es Study.* European Journal of Internal Medicine **102,** 80-87 (2022)
37. Nii, M., et al. *Medical Checkup and Image Data Analysis for Preventing Life Style Diseases: A Research Survey of Japan Society for the Promotion of Science with Grant-in-Aid for Scientific Research (A) (Grant number 25240038)*. in *2015 7th International Conference on Emerging Trends in Engineering & Technology (ICETET) (*2015)
38. Deberneh, H.M. and I. Kim, *Prediction of Type 2 Diabetes Based on Machine Learning Algorithm.* International Journal of Environmental Research and Public Health **18**(6), 3317 (2021)
39. Wu, Y., et al., *Machine Learning for Predicting the 3-Year Risk of Incident Diabetes in Chinese Adults.* Frontiers in Public Health **9** (2021)
40. Hahn, S.-J., et al., *Prediction of type 2 diabetes using genome-wide polygenic risk score and metabolic profiles: A machine learning analysis of population-based 10-year prospective cohort study.* Ebiomedicine **86,** 104383 (2022)
